# Hierarchical, Grid-Aware, and Economically Optimal Coordination of Distributed Energy Resources in Realistic Distribution Systems

**DOI:** 10.3390/en13236399

**Published:** 2022

**Authors:** Mads Almassalkhi, Sarnaduti Brahma, Nawaf Nazir, Hamid Ossareh, Pavan Racherla, Soumya Kundu, Sai Pushpak Nandanoori, Thiagarajan Ramachandran, Ankit Singhal, Dennice Gayme, Chengda Ji, Enrique Mallada, Yue Shen, Pengcheng You, Dhananjay Anand

**Affiliations:** 1 Department of Electrical and Biomedical Engineering, University of Vermont, Burlington, VT 05405, USA; 2 Pacific Northwest National Laboratory, Electricity Infrastructure and Buildings Division, Richland, WA 99352, USA; 3 Whiting School of Engineering, Johns Hopkins University, Baltimore, MD 21218, USA; 4 National Institute of Standards and Technology, Smart Grid Program, Gaithersburg, MD 20899, USA

**Keywords:** distributed energy resources, smart loads, flexibility, distribution system operator, distribution network, optimal power flow, control, large scale, solar energy

## Abstract

Renewable portfolio standards are targeting high levels of variable solar photovoltaics (PV) in electric distribution systems, which makes reliability more challenging to maintain for distribution system operators (DSOs). Distributed energy resources (DERs), including smart, connected appliances and PV inverters, represent responsive grid resources that can provide flexibility to support the DSO in actively managing their networks to facilitate reliability under extreme levels of solar PV. This flexibility can also be used to optimize system operations with respect to economic signals from wholesale energy and ancillary service markets. Here, we present a novel hierarchical scheme that actively controls behind-the-meter DERs to reliably manage each unbalanced distribution feeder and exploits the available flexibility to ensure reliable operation and economically optimizes the entire distribution network. Each layer of the scheme employs advanced optimization methods at different timescales to ensure that the system operates within both grid and device limits. The hierarchy is validated in a large-scale realistic simulation based on data from the industry. Simulation results show that coordination of flexibility improves both system reliability and economics, and enables greater penetration of solar PV. Discussion is also provided on the practical viability of the required communications and controls to implement the presented scheme within a large DSO.

## 1. Introduction

### 1.1. Motivation

For a century, distribution system operators (DSOs) have managed a system wherein power flowed from large, central thermal generators in high voltage (HV) transmission networks to medium voltage (MV) primary distribution networks to low-voltage (LV) secondary distribution networks where loads consumed energy. In this paradigm, the timescales of aggregate loads were so slow that monthly customer meter readings were sufficient for reliable grid operations. However, the last decade has seen a precipitous drop in solar photovoltaic (PV) costs [1], which together with aggressive renewable portfolio standards and public demand for clean energy has led to increasing deployments of variable and distributed generation in distribution networks. In some US states, such as California, Hawaii, Vermont, and New York, extreme levels of solar PV generation (e.g., >50% of annual demand supplied by solar PV) already represent a fundamental engineering challenge for electric distribution system operations and will require a much more flexible electricity grid [2]. Specifically, the energy storage capability inherent to many end-use appliances is expected to underpin a flexible demand that can reduce curtailment of renewable generation and support active distribution network operations [3,4].

Distribution feeders with the expected MWs of solar PV and flexible demand represent a grid that interacts with thousands of controllable inverters and kW-scale loads, such as thermostatically controlled loads (e.g., electric water heaters, residential air-conditioners), deferrable loads (e.g., electric vehicle chargers, smart appliances), and distributed batteries. These “future” systems are already being enabled by cheap “printable” embedded hardware platforms, such as the Internet of Things (IoT), and people’s desire for comfort and convenience that are opening up a new frontier for energy digitization [5]. Indeed, as live sensing, connectivity, and computing become inexpensive, they become ubiquitous. That is, energy technology is advancing faster than the electricity infrastructure around it. Thus, there is a need to reconsider the role of the distribution system operators (DSOs) as solar PV and smart inverters are increasingly deployed and demand becomes flexible.

### 1.2. Related Literature

While it has been clear for some time that DSOs need to evolve from passive/reactive network managers to active network operators [6–10], it has been less clear how a DSO should manage the influx of thousands of connected, controllable devices (e.g., PV inverters and smart appliances), particularly in the face of changing grid and wholesale market conditions. The required coordination between a DSO’s network and many DER owners and aggregators will become critically important at scale. The need to address these challenges has spurred a multitude of advanced concepts and models for how DSOs can interact with DERs, aggregators, and wholesale (transmission) markets [11,12]. One popular approach is the so-called “transactive energy” paradigm, where market-centric schemes can engender holistic (TSO-DSO-Aggregator) coordination of DERs by broadcasting market price signals to devices and devices managing price sensitivities [13]. However, with large-scale participation of DERs, transactive energy can be susceptible to harmful load synchronization effects, power oscillations, and volatile prices–especially when distribution circuits constrain DER behaviors–as shown in [14].

Thus, any DER coordination scheme should carefully consider distribution circuits, which represent data owned and managed by the utility (or DSO) and is the reason why this manuscript focuses on the so-called “Market DSO” model, e.g., see [11]. In the Market DSO model, the DSO performs all coordination, aggregation, and control of DERs to deliver grid services across different timescales. While such a DSO-centric model could preclude independent DER aggregators (i.e., increases regulatory complexity), the model simplifies the role of wholesale market signals (i.e., independent or transmission system operators, ISO or TSO), to include interactions with only large aggregated DSO resources and thereby eliminates the need for these markets to be cognizant of distribution network conditions or individual DER owners or aggregators. This Market DSO model is similar to innovative energy service provider models proposed by entities such as Consolidated Edison Company of New York [15]. Within the context of the utility-centric Market DSO, any DER coordination scheme must holistically integrate market signals, multi-phase AC networks, and device signals in a manner that is scalable across the appropriate spatial and temporal scales.

Thus, DER coordination under the Market DSO model should effectively account for AC network constraints, which has recently been termed “grid-aware” coordination (as opposed to grid-agnostic coordination) [16]. Grid-aware coordination of DERs has often employed optimization-based methods, such as [17], where the aggregator’s DER control signals track a Karush-Kuhn-Tucker (KKT) point that satisfies the KKT optimality conditions. However, for the non-convex AC OPF, the KKT conditions may not be sufficient to guarantee global optimality. Other optimization schemes can provide market services using “virtual batteries” (VBs) without exact grid models nor real-time measurements [18]. However, these methods do not directly incorporate multi-period energy constraints and the KKT point can be sensitive to exogenous disturbances, which we overcome in this manuscript by explicitly computing an optimal power flow (OPF) solution that is feasible with respect to underlying nonlinear AC physics and provides an upper bound on optimality gap. To overcome the effect of disturbances while tracking an aggregate power signal, the literature has recently focused on real-time control of DERs in microgrid settings [19]. These works generally consider using droop characteristics to generate active and reactive power set-points for DERs with local measurements of frequency and voltage and compensating for the deviations. However, the local controller design often is not cognizant of network-wide conditions nor economic signals or desired power trajectories. While [20] develops a local (proportional) controller that incorporates network parameters and conditions into controller gains to minimize voltage deviations with active power injections, it does not consider system-wide power tracking objectives such as an economic trajectory that satisfies voltage limits across the feeder. Economics and network-wide controller gains are presented in [21], where a distributed averaging PI (DAPI) control strategy is used to ensure proportional power-sharing and economic optimality. However, since it requires extensive communication between the DERs, it may not be feasible on a larger geographic scale. Moreover, while the droop coefficients in [21] are chosen proportionally according to DER power limits, state-of-charge limits are not considered, and the coefficients are not optimized to minimize head node power deviations from the economic trajectory. In this current manuscript, we overcome the above challenges associated with real-time control of DERs by employing a hierarchy that leverages a utility’s SCADA, network data, available real-time sensors, and the DERs’ energy states to update local controller gains and update set-points, so they are cognizant of grid and energy conditions. That is, this manuscript presents one possible realization of the Market DSO by systematically coordinating TSO market interactions, AC network constraints and physics, and DER capabilities through a novel hierarchical control scheme that is practically implementable. Next, we summarize the proposed hierarchy.

### 1.3. Summary of Proposed Research Contributions

**Figure 1.**
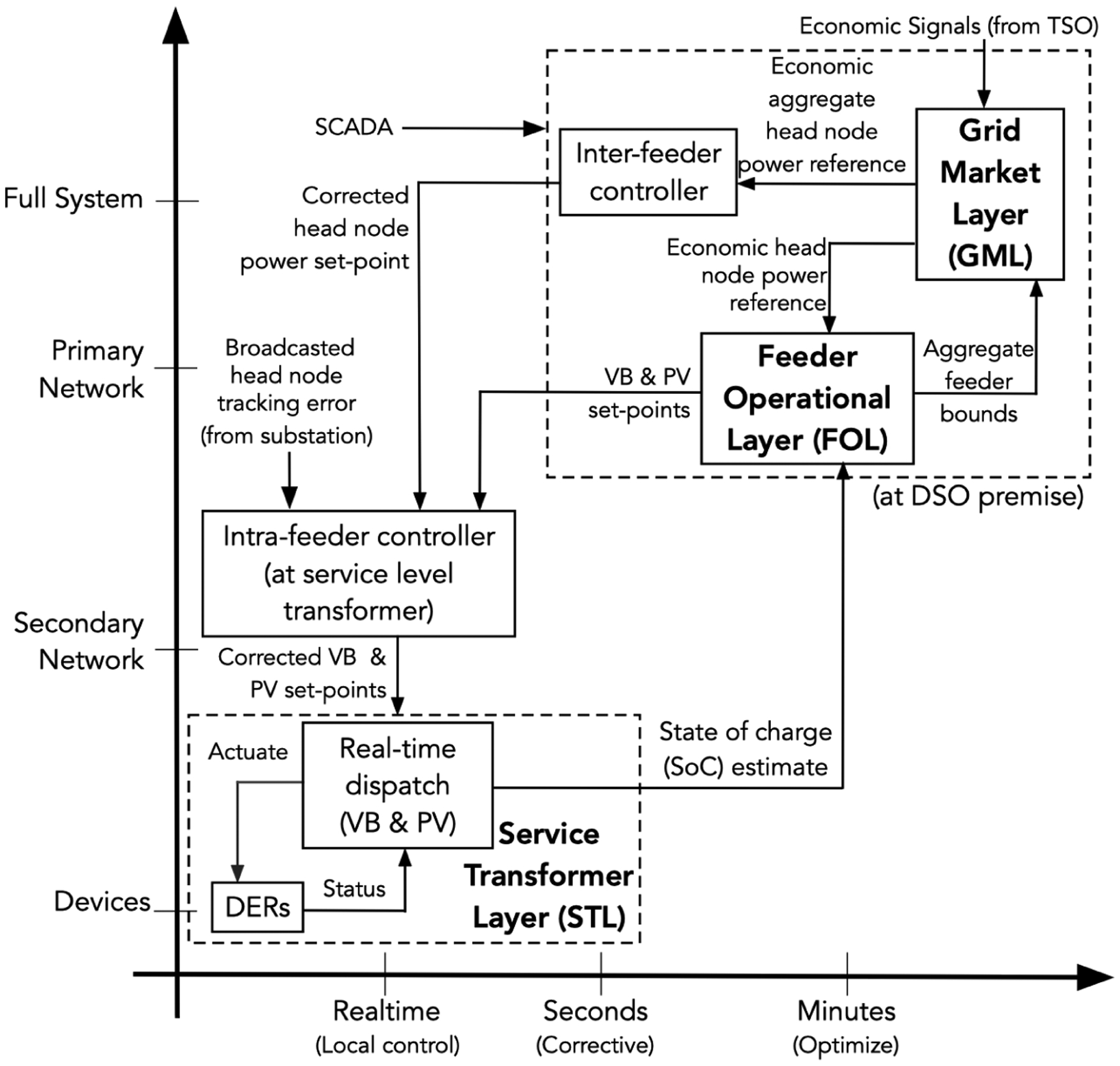
Hierarchical distributed energy resources (DER) control scheme along qualitative spatio-temporal scales.

The presented three-layer hierarchical DER control scheme adapts spatio-temporal concepts from conventional frequency control in transmission systems to a new approach for power regulation in distribution systems and is summarized in Figure 1. In particular, we adopt the three-layer type model of conventional frequency control, in which primary control is performed by local droop controllers in real-time (sub-second) while secondary control balances regional areas on a timescale of 30 s to 90 s, and tertiary control represents economic wholesale market clearing mechanisms for the entire system that are updated every 5 min to 15 min and scheduled hourly. Together, these three layers balance supply and demand to ensure tight control of frequency in bulk power systems. The presented work, analogously separates layers through timescale and spatial separation in distribution systems. The first layer controls DERs at each service transformer in real-time to track a power reference and is called the service transformer layer (STL). The power reference is provided at each STL element by the feeder operational layer (FOL), which optimizes feeder-wide operations to track a power set-point at the distribution substation. The set-point at every substation is provided by the TSO-DSO interface called the grid market layer (GML), which minimizes costs across all the DSO’s interconnected substations.

Thus, the STL is tasked with performing local real-time control of a small groups of DERs (e.g., solar PV inverters and smart appliances) every few seconds to manage power exchanges at the service transformer (i.e., the interface between primary and secondary distribution networks). Due to the local nature of control enabled by the hierarchical design, the STL controller has access to the static parameters (e.g., rated power), sensor measurements and control inputs of the DERs connected to the same service transformer node. Each STL controller is tasked with: (a) updating an aggregated dynamic flexibility model for the DERs; and (b) real-time dispatch of the DERs to track certain power set-points. In particular, each STL controller constructs a dynamic representation of energy and power flexibility limits for the group of DERs at the service transformer, that together is denoted by a VB model [22–26]. The VB’s power limits represent the (maximal) range of the control set-points that can be successfully tracked by the DERs at each service transformer; while VB’s energy limits encode the end-user quality of service constraints and, along with the estimated state of charge (SoC), determine the duration of successful tracking performance. Different methods exist for characterizing the VB model of an aggregation of DERs, including closed-form expressions [22], optimization-based methods [23,25], as well as deep learning techniques [24,26]. Finally, the STL controller performs a real-time optimal control of the DERs (e.g., switching thermostatic loads on/off) to track the power set-points by explicitly accounting for service transformer and DER quality of service constraints, as necessary [25]. The STL, therefore, represents groups of DERs in the secondary distribution network as dispatchable PV inverters and VBs in each phase of the primary distribution network.

The second layer in the hierarchy is the FOL, which employs a three-phase distribution network model of the primary network (i.e., an unbalanced feeder) to optimize PV and VBs’ power set-points every 1 min to 5 min and ensure voltage and current limits are satisfied [27–29]. The objective of the FOL is to coordinate the flexibility of the responsive VBs and PV inverters with the (mechanically actuated) legacy control devices, such as capacitor banks (CBs) and on-load tap changers (OLTCs), to reshape net power exchanges at the feeders’ head-nodes (i.e., at the distribution substations) in response to economically optimized power set-points provided by the GML, which updates every five minutes and represents the top level of the hierarchy.

The GML represents the DSO’s scheduling coordinator at the interface between the MV distribution system and the TSO’s market and converts market signals into optimized power set-points at the distribution substation of every feeder. Since the flexibility inherent to VBs is energy-constrained and the feeders are networked within a DSO’s large sub-transmission system, the GML considers a multi-period model of a simplified sub-transmission network where every feeder is represented as a PQ-load with controllable aggregated VB and PV inverter resources. In this work, the GML optimizes resources for economic benefits from peak demand reduction, arbitrage across the TSO’s day-ahead and real-time markets as well as balancing reserve provisioning from ancillary markets.

Together, the three layers (GML, FOL, and STL) mirror the voltage hierarchy of the DSO’s HV, MV, and LV system interfaces, which aligns with the Market DSO model described above and enables a scalable approach to manage millions of controllable DERs across a DSO’s entire system. Besides, the alignment permits utilization of a DSO’s secure and low-latency communication network between sub-transmission nodes (i.e., GML elements), distribution substation nodes (i.e., FOL elements), and service transformer nodes (i.e., STL elements). Specifically, we leverage the communication system to design and employ a proportional intra-feeder control scheme that provides sub-second updates to the FOL’s VB power set-points to correct for forecast errors and model mismatch. Furthermore, to account for model mismatch in the GML, the DSO’s SCADA system is employed to provide sub-minute inter-feeder corrective updates to the GML’s economic power set-points of feeder head-nodes. The key contributions of this manuscript include the following:

- The presented GML-FOL-STL-DER hierarchical scheme represents a novel, scalable, and practically implementable approach to the Market DSO’s task of coordinating DERs while accounting for individual device and AC grid constraints;
- The scheme employs optimization-based methods within each layer to ensure that DERs are utilized optimally and in a “grid-aware” manner, and then integrates the layers with feedback-based control schemes to be robust against model-mismatch and forecast errors.
- Simulation-based analysis is conducted based on realistic network models from a New York DSO which validates the coupled GML-FOL-STL operations and highlights the role and value of the proposed hierarchical scheme.

The rest of the manuscript is organized as follows: Section 2 describes salient system considerations related to market signals and services, unbalanced AC physics (i.e., grid signals), and DER models and considerations (i.e., device signals). An overview of the hierarchical scheme along with the role, inputs, outputs of each layer is presented in Section 3. Section 4 presents the GML’s market-based iterative optimization formulation and provides a peak-shaving example. The FOL’s computationally-efficient, robust OPF formulation is presented in Section 5 together with an illustrative example. Section 6 describes a fast optimization-based algorithm for dispatching DERs while accounting for device-level constraints. In Section 7, the proposed inter-layer communications and controls are presented and real-time practicalities are discussed along with proof of concept examples. A large-scale, realistic test system is presented in Section 8 and simulation-based analysis is conducted for the coupled GML-FOL-STL hierarchy that represents the Market DSO model. Finally, Section 9 concludes the paper with a summary of results and a discussion of future research directions.

## 2. System Models and Consideration

The proposed hierarchical scheme converts system-wide economic market signals into power reference signals for the DSO’s feeders, which achieve their reference by managing devices internal to each feeder. Thus, each layer is defined by key signals that are described next.

### 2.1. Market Signals for the GML

The GML manages the coordinated participation of aggregated DERs in both wholesale energy and ancillary service markets. The energy market usually operates in a two-settlement manner and clears transactions at two timescales with respective prices, i.e., a day ahead with day-ahead/forward price and real-time, usually 5 min ahead, with real-time/spot price. We assume the clearing times of the ancillary service market coincide with the real-time market on a 5-minute basis, enabling co-optimized cross-market participation. Further, the GML is assumed to be a price taker, i.e., has no significant effect on prices.

#### Day-Ahead Market:

The day-ahead market runs for each hour of the next day simultaneously. The GML determines *optimal hourly procurement of energy* in the day-ahead market based on *predicted hourly prices* and feeder specifications subject to uncertainties in the day-ahead forecast. In light of the potential price differences and the chance for arbitrage across stages, the GML may indeed predict *two-stage price differences* and over- or under-procure energy to minimize net cost.

#### Real-Time Market:

**Figure 2.**
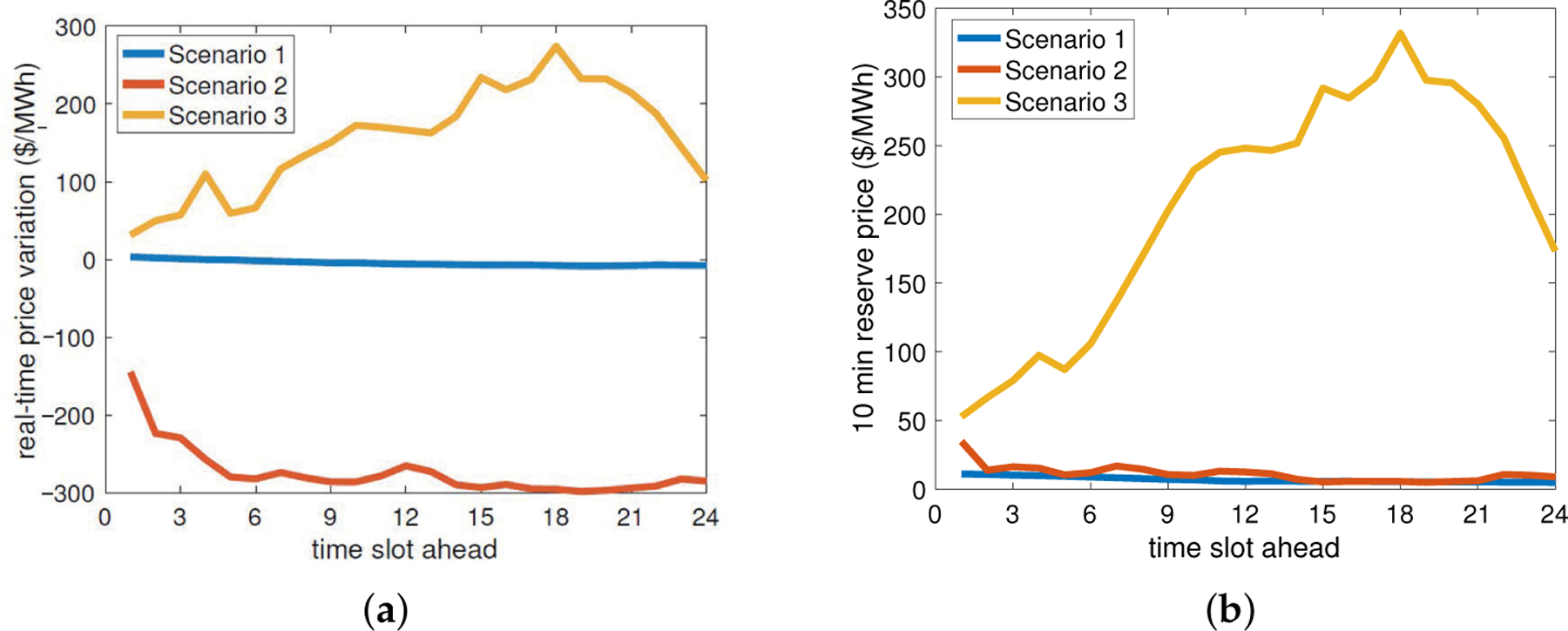
An example of scenario clustering for real-time price changes and reserve prices. (**a**) Real-time price change scenarios. (**b**) Reserve price scenarios.

The real-time market runs to offset any deviation from the day-ahead schedule for each 5-minute slot before actual operation. This rolling market-clearing implies that the GML has the chance to update its *commitment to procure energy* in the real-time market for the next slot with the latest information, e.g., *real-time price predictions*, after observing the market outcome of the current slot. Indeed, the GML uses a scenario-based approach to predict future prices based on a set S of dominant scenarios of price changes, i.e., $\Delta \lambda_{s}^{rt}(\tau )$, *τ* = 1, 2, …, *T*^*pred*^, s∈S, with respect to the latest price realization, where *T*^*pred*^ is the length of prediction. For instance, at time *t*, given the latest real-time price *λ*^*rt*^(*t* − 1), the forthcoming prices are predicted as $\lambda_{s}^{rt}(t-1+\tau )≔\lambda^{rt}(t-1)+\Delta \lambda_{s}^{rt}(\tau )$, *τ* = 1, 2, …, *T*^*pred*^, s∈S. These scenario-based predictions are extracted from NYISO-based real-time prices [30], and Figure 2a illustrates an example of 3 scenarios of price changes with the prediction length of 2 h for a particular time of a day.

#### Ancillary Service Market:

We will specifically focus on the ancillary service of 5-min operating reserves from NYISO. The GML, while participating in the real-time market, can simultaneously provide reserve service by tracking designated commands within a committed bound/capacity. Likewise, it can manage and update *reserve commitments* 5 min ahead using the latest information, e.g., *reserve price predictions*, due to the repeatedly rolling market operation. The GML obtains the future reserve price predictions *α*_*s*_(*τ*), *τ* = 1, 2, …, *T*^*pred*^, s∈S, using the same scenario-based approach, except that the scenarios are directly defined on reserve prices rather than price changes. Note that due to the strong coherence between real-time prices and reserve prices, the scenarios s∈S are clustered jointly based on data from NYISO historical data. Figure 2b shows an example of reserve price scenarios.

#### Peak Demand Charge:

We include a GML mode that accounts for peak demand charges, which assigns a large cost to the peak demand occurring during a specified period, e.g., a month. This one-time payment comprised of a unit price way higher than average energy prices, e.g., for a NY utility, the is price *γ* = $10, 000/MW per month. The GML peak shaving mode allows utilities to use this mode to reduced this significant expenditure.

### 2.2. Grid Signals for the FOL

The market signals shape the GML’s power reference signals at each distribution substation (i.e., head-node), which the FOL uses to coordinate each feeder’s STL elements. This coordination requires that the FOL manages physical grid signals, such as voltage and current phasors that are representative of unbalanced distribution feeders. Thus, the aim of this section is to develop an AC power flow model of an unbalanced distribution feeder that relates the feeder’s voltage and current signals with power injections. Specifically, we employ a branch flow model (BFM) to represent the AC physics in the unbalanced feeder [27].

#### 2.2.1. Modeling Unbalanced Feeders

In modeling 3-phase AC power flows, we need to leverage mathematical operators |.|, ◦, (.)* and diag(.) to represent the cardinality of a set, the Hadamard product of matrices, the complex conjugate operator, and the diagonal operator, respectively. Then, given a radial, 3-phase feeder with *N* nodes, denote N={1,2,…,N} as the set of all nodes, *ϕ* = {*a*, *b*, *c*} as the set of phases at each node, L={1,2,…,L}={(m,n)}⊂(N×N) as the set of *L* branches, and G={1,2,…,G}⊆N as the set of all nodes with DERs. Let vector $V_{n}(t)\in {\mathbb{C}}^{\left|\phi \right|}$ be the complex voltage at node *n* and time *t*, with *W*_*n*_(*t*) = *V*_*n*_(*t*)*V*_*n*_(*t*)*, $i_{l}(t)\in {\mathbb{C}}^{\left|\phi \right|}$ be the current in branch *l* at time *t*, with *I*_*l*_(*t*) = *i*_*l*_(*t*)*i*_*l*_(*t*)*, *S*_*l*_(*t*) = *V*_*n*_(*t*)*i*_*l*_(*t*)* be the apparent power in branch *l* at time *t*. Further, let $Z_{l}=R_{l}+jX_{l}\in {\mathbb{C}}^{\left|\phi |\times |\phi \right|}$ be the impedance matrix of branch *l*. Let $S_{n}^{\text{net}}(t)\in {\mathbb{C}}^{\left|\phi \right|}$ be the complex net power injection at node *n* at time *t* and is based on complex solar PV inverter injections and electric demand, $S_{n}^{\text{S}}(t)$, $S_{n}^{\text{L}}(t)\in {\mathbb{C}}^{\left|\phi \right|}$, respectively. In addition, let $P_{n}^{\text{b}}(t)\in {\mathbb{R}}^{\left|\phi \right|}$ be the active power delivered from a battery at node *n* at time *t*. Herein, we assume that each resources is connected to a single phase. Based on the above notation, the physics of 3-phase AC power flows are given by the following equations:

(1) $$0=W_{n}(t)-W_{m}(t)+\left(S_{l}(t)Z_{l}^{*}+Z_{l}S_{l}(t)\right)-Z_{l}I_{l}(t)Z_{l}^{*}\;\forall l\in L$$

(2) $$0=\text{diag}\left(S_{l}(t)-Z_{l}I_{l}(t)-\sum_{p}S_{p}(t)\right)+S_{n}^{\text{net}}(t)\;\forall l\in L$$

(3) $$\left[\begin{array}{cc} W_{n}(t) & S_{l}(t)\\ S_{l}{\left(t\right)}^{*} & I_{l}(t) \end{array}\right]=\left[\begin{array}{c} V_{n}(t)\\ i_{l}(t) \end{array}\right]{\left[\begin{array}{c} V_{n}(t)\\ i_{l}(t) \end{array}\right]}^{*}\;\forall l\in L$$

(4) $$0=\text{real}\left\{S_{n}^{\text{net}}(t)-S_{n}^{\text{S}}(t)+S_{n}^{\text{L}}(t)\right\}-P_{n}^{\text{b}}(t)\;\forall n\in G$$

(5) $$0=\text{imag}\left\{S_{n}^{\text{net}}(t)-S_{n}^{\text{S}}(t)+S_{n}^{\text{L}}(t)\right\}\;\forall n\in G,$$

where (1) relates the voltage drop in the network with the branch power flows, (2) represents the power balance equation at each node which ensures that the power entering a node equals the power leaving, and (3) is the non-linear power flow constraint that relates voltages and currents to new matrix variables *W*_*n*_(*t*), *I*_*l*_(*t*) and *S*_*l*_(*t*). In (4) and (5), the active and reactive net nodal power injections $S_{n}^{\text{net}}(t)$ are defined in terms of solar $S_{n}^{\text{S}}(t)$, load $S_{n}^{\text{L}}(t)$, and battery $P_{n}^{\text{b}}(t)$ injections.

### 2.3. Device Signals

A significant portion of the building load can be attributed to thermostatic loads, such as air-conditioners (ACs), comprising approximately 42% of total energy usage and 44% of peak energy usage (14:00 to 20:00) in the U.S., and water-heaters (WHs), with the corresponding numbers as 10% and 9%, according to a recent report by the U.S. Department of Energy [31]. As such, in this paper, we focus our attention on ACs and WHs as use-case examples of flexible loads for illustration of the STL modeling and control algorithms. Thermostatic loads such as residential ACs and WHs are a form of switching loads whose power consumption toggles between two discrete operational states (‘on’ and ‘off’) in order to maintain certain end-use specified thermal constraints. Normal operation of these thermostatic loads is described by the following hybrid dynamical system models (6) and (7).

#### AC Model:

The operation of a residential AC is governed by thermal dynamics of the room temperature, as represented by [32,33],

(6) $$\dot{T}(t)=-\frac{\left(T(t)-T_{a}\right)}{CR}-\frac{\eta p(t)}{C},\;p\left(t^{+}\right)=\{\begin{array}{cc} 0, & \text{if}\;T(t)\leq T_{set}-\delta T/2\\ P, & \text{if}\;T(t)\geq T_{set}+\delta T/2\\ p(t), & \text{otherwise} \end{array},$$

where *T*(*t*) is the room temperature; *p*(*t*) ∈ {0, *P*} represents the power withdrawal of the AC; *T*_*a*_ denotes the outside air temperature; and *C*, *R*, *η* are the device parameters representing the room thermal resistance, thermal capacitance and the load efficiency, respectively. *T*_*set*_ is the temperature set-point and *δT* represents the width of the temperature hysteresis deadband.

#### WH Model:

The operation of an electric WH is governed by the thermal dynamics of the water temperature. In the simplistic ‘one-mass’ thermal model which assumes that the temperature inside the water-tank is spatially uniform (valid when the tank is *nearly* full or *nearly* empty) [33–35], the water temperature dynamics can be expressed in the form of:

(7) $$\begin{array}{l} {\dot{T}}_{w}(t)=\frac{\dot{m}C_{p}\left(T_{in}-T_{w}(t)\right)+W\left(T_{a}-T_{w}(t)\right)}{C_{w}}+\frac{p(t)}{C_{w}}\\ p\left(t^{+}\right)=\{\begin{array}{cc} P, & \text{if}\;T_{w}(t)\leq T_{set}-\delta T/2\\ 0, & \text{if}\;T_{w}(t)\geq T_{set}+\delta T/2\\ p(t), & \text{otherwise} \end{array}, \end{array}$$

where *T*_*w*_(*t*) denotes the temperature of the water in the tank; *p*(*t*) ∈ {0, *P*} represents the power draw of the WH; *T*_*set*_ is the temperature set-point of the WH with a deadband width of *δT*; *T*_*a*_ denotes the room temperature; *T*_*in*_ is the inlet water temperature; *ṁ* is hot water flow rate; *C*_*w*_ is the thermal capacitance of the water in the tank; *C*_*p*_ is the specific heat capacity of the water; and *W* is the thermal conductance of the tank shell.

In this work, we assume that the DER coordinator is able to toggle the operational state of a small fleet of thermostatic loads (by appropriately switching those on/off) via control commands, and thereby change the aggregated power consumption to track certain power set-points at the service transformer. Due to the thermal dynamics, such changes in operational states of the flexible thermostatic loads do not lead to immediate perceptible changes in the associated (room/water) temperature variable—allowing for some flexibility in power consumption over a finite duration of time.

#### VB Model:

This flexibility can be represented by a virtual battery (VB) model [22–26] that captures the power (response) and energy (duration) limits on the aggregate control offered by the DERs. A VB is typically modeled in the form of first-order dynamics to represent the temporal evolution of the virtual energy state driven by changes in the power consumption as a control input, with constraints specified on the power set-points and the (virtual) energy states [22,24,26]. In this work, for illustrative purpose, we present a deterministic VB model with closed-form expressions for its parameters, leveraging full DER information from the local controller to reasonably assume availability of required device-specific parameters of all the DERs. Thus, consider an aggregation of *N* thermostatic loads behind a service transformer, where each thermostatic load is indexed by *i* = 1, …, *N*. Note that the hybrid dynamical model of the *i*-th thermostatic load, described by either (6) or (7), can be compactly represented in the following generalized form [22–24]:

(8) $${\dot{B}}_{i}(t)=-a_{i}B_{i}(t)-P_{i}^{\text{b}}(t),\;B_{i}(0)=B_{0,i}\;B_{i}(t)\in \left[B_{\text{min},i}(t),B_{\text{max},i}(t)\right]\;P_{i}^{\text{b}}(t)\in \left[P_{\text{min},i}(t),P_{\text{max},i}(t)\right]$$

for some *B*_*i*_(*t*) considered as the ‘virtual’ state of charge (SoC) with *B*_0,*i*_ as its initial condition; *a*_*i*_ as the self-dissipation rate (typically small); $P_{i}^{\text{b}}(t)$ as a control input denoting the power injection (into the grid) above a nominal (or, baseline) power profile; *B*_min_ and *B*_max_ denote the lower and upper energy limits, respectively; while *P*_min_ and *P*_max_ are the lower and upper power limits. Equations of the form (8) represent a VB model of device *i*. Note that we use the notion of injected power (as opposed to consumed power) to align with the typical grid modeling convention of treating power injected into the grid as positive (while any power consumed is assigned a negative value). Following this convention, we can use the example of the AC to briefly explain how to obtain (8) from (6). Specifically, we define the ‘baseline’ power injection (*p*_*base*,*i*_ < 0) of an AC as the negative of the time-average of the power consumption when the room temperature is maintained close to the desired temperature set-point. From (6), we have *p*_*base*,*i*_ = (*T*_*set*,*i*_−*T*_*a*_)/*η*_*i*_*R*_*i*_, where the sub-script *i* is used for the *i*-th load. The rest follows by defining a new state variable (or, virtual SoC) as *B*_*i*_(*t*) ≔ *C*_*i*_ (*T*_*set*,*i*_−*T*_*i*_(*t*))/*η*_*i*_, and the control input as $p_{i}^{\text{b}}(t)≔-p_{i}(t)-p_{\text{base},i}$. Thus, device *i* has a corresponding VB model of the form (8) and can be parameterized by Φ_*i*_ ≔ (*a*_*i*_, *B*_min,*i*_, *B*_max,*i*_, *P*_min,*i*_, *P*_max,*i*_, *B*_0,*i*_). This device-level description of flexibility is used as a building block to characterize flexible demand from *N* devices behind each service transformer.

While there are various data-driven methods for computing the aggregated VB parameters [23–26,36], we adopt the closed-form expressions proposed in [22], for illustration in this paper. Typically, the value of the self-dissipation coefficients (*a*_*i*_) are very small. Therefore, under the simplifying assumption of *a*_*i*_ ≈ *a*, ∀*i*, we define the aggregated virtual SoC as $B≔\sum_{i=1}^{N}B_{i}$ and the aggregated control input as $P^{\text{b}}≔\sum_{i=1}^{N}P_{i}^{\text{b}}$, and obtain the aggregated VB model as [22]:

(9) $$\dot{B}(t)=-aB(t)-P^{\text{b}}(t),\;B(0)=B_{0}\;B(t)\in \left[B_{\text{min}}(t),B_{\text{max}}(t)\right]\;P^{\text{b}}(t)\in \left[P_{\text{min}}(t),P_{\text{max}}(t)\right]$$

Thus, this aggregated VB is represented by

$$\Phi =\left(a,\sum_{i=1}^{N}B_{\text{min},i},\sum_{i=1}^{N}B_{\text{max},i},\sum_{i=1}^{N}P_{\text{min},i}^{\text{b}}\sum_{i=1}^{N}P_{\text{max},i}^{\text{b}},\sum_{i=1}^{N}B_{0,i}\right).$$

Note that the aggregated VB model (9) guarantees that for every set of ‘admissible’ trajectories $P_{i}^{\text{b}}(t)$ for each of individual DER *i* = 1, …, *N*, the aggregated power trajectory *P*^b^(*t*) is also admissible at the service transformer level.

## 3. Overview of Hierarchical DER Control Scheme

In this section, we expand on the high-level summary provided in Section 1.3 and discuss the roles of the different layers and the inputs and outputs that are used for coordinating GML-FOL, FOL-STL, and STL-DER interfaces. Specifically, the proposed spatio-temporal decomposition of the DSO’s grid operations enables scalable coordination of the market, grid, and device signals from Section 2 to engender the relevant set-points within each layer. Figure 1 illustrates the spatio-temporal decomposition and corresponding inputs and outputs, which are discussed next.

- **Grid market layer (GML)** employs a TSO’s market signals to optimize the dispatch of available, aggregated flexibility from all feeders and deliver economically optimal power set-points for each feeder’s headnode in the DSO’s system. Since we use market signals from New York’s TSO (NYISO), we consider the GML on a timescale of 5 minutes, which matches the update rate of NYISO’s “real-time market.”
  - Input: market signals (from TSO); bounds on flexibility for aggregated feeders (from FOL)
  - Output: economic feeder power reference (to FOL)
- **Feeder operational layer (FOL)** employs the GML’s desired power reference trajectory at each headnode, the DSO’s unbalanced distribution network models, and the STL’s VB model to optimize the dispatch of controllable assets within a feeder so as to minimize power deviations from the headnode reference. The controllable assets include groups of DERs (i.e., a VB) and PV inverters that together track the GML’s economic power reference at the feeder’s head-node while maintaining an acceptable voltage profile throughout the feeder. Since the FOL responds to forecast errors and that solar PV variability is on the order of minutes, the FOL’s timescale has been selected as 1 min.
  - Input: economic feeder head-node power reference (from GML); VB model parameters and VB state of charge (from STL)
  - Output: bounds on flexibility for aggregated feeders (to GML); VB power set-points (to STL)
- **Service transformer layer (STL)** employs the FOL’s optimal resource dispatch signal at each (primary) node in the feeder along with DER data to coordinate small, local groups of DERs while accounting for local device constraints on power and energy (e.g., temperature bounds prescribed by users). Since we need to update the DER dispatch often to reject any un-modeled disturbances (e.g., inflexible, background demand), we have selected a timescale of 1 s for the STL’s dispatch loop.
  - Input: VB power set-points (from FOL); DER data (from DER)
  - Output: updated VB state of charge estimate (to FOL); DER control signal (to DER)

The next three sections describe the GML (Section 4), FOL (Section 5), and STL (Section 6) elements in detail, including their optimization-based formulations along with an illustrative example of each individual layer.

## 4. Grid Market Layer (GML)

The GML optimizes flexibility of distribution-level aggregated DERs to jointly participate in the transmission-level energy and ancillary service markets, which determines the optimal aggregate interactions with wholesale energy and ancillary markets as well as feeder-level set-points for DERs to track. We first present the operational constraints of feeder-level virtual batteries, solar generation, bank-level transformers, and reserve commitments. For computational tractability, we then adopt a linearized power flow model for the distribution network, which is not necessarily radial, and calibrate its power flow solution through a three-layer tuning mechanism. The system uncertainty in market prices is handled with a scenario-based approach and a receding horizon implementation framework to alleviate its impact. This GML model is demonstrated in a real-world NYISO system with 212 feeders and 79 banks.

### 4.1. Operational Constraints

#### Feeder Level:

Each feeder is modeled by approximation as an ensemble of a feeder-level solar PV inverter, a VB, and a fixed amount of inelastic demand. We use $P_{f}^{g}(t)$ and $Q_{f}^{g}(t)$ to denote the controlled real and reactive solar power generation at the feeder *f* at time *t*, respectively, subject to availability. The apparent power each feeder can supply is further constrained by the inverter capacity $S_{f}^{\text{max}}(t)$, given by

(10) $$P_{f}^{\text{min}}(t)\leq P_{f}^{g}(t)\leq P_{f}^{\text{max}}(t),$$

(11) $$\left|Q_{f}^{g}(t)\right|\leq \eta_{PF}P_{f}^{\text{max}}(t),$$

(12) $${\left(Q_{f}^{g}(t)\right)}^{2}+{\left(P_{f}^{g}(t)\right)}^{2}\leq {\left(S_{f}^{\text{max}}(t)\right)}^{2},$$

where *η*_*PF*_ specifies a limiting power factor. The VB at feeder *f* is characterized by its charge/discharge dynamics, bounded by its capacity and power rating:

(13) $$B_{f}(t+1)=B_{f}(t)-\delta_{t}R_{f}(t)+W_{f}(t),$$

(14) $$B_{f}^{\text{min}}(t)\leq B_{f}(t)\leq B_{f}^{\text{max}}(t),$$

(15) $$R_{f}^{\text{min}}(t)\leq R_{f}(t)\leq R_{f}^{\text{max}}(t),$$

where *B*_*f*_(*t*) denotes the VB state of charge and *R*_*f*_(*t*) denotes its discharge rate at time *t*. Note that a negative *R*_*f*_(*t*) indicates charging. *W*_*f*_(*t*) represents exogenous change in the VB stage of charge due to a storage component resource such as an EV disconnecting from a feeder. *δ*_*t*_ is the time interval of each slot.

#### Bank Level:

The secondary feeders are connected to transformer banks. We use B to denote the set of banks, a.k.a. buses, in the distribution network with indices i=1,2,⋯,|B|, and $F_{i}$ to denote the set of secondary feeders connected to bank *i*, with $F≔\cup_{i\in N}F_{i}$ being the set of all feeders in the distribution network, and L to denote the set of lines that connect transformer banks. The real and reactive net power withdrawal *P*_*i*_(*t*), *Q*_*i*_(*t*) on transformer bank *i* at time *t* is given by

(16) $$P_{i}(t)=\sum_{f\in F_{i}}\left(P_{f}^{d}(t)-P_{f}^{g}(t)-R_{f}(t)\right),$$

(17) $$Q_{i}(t)=\sum_{f\in F_{i}}\left(Q_{f}^{d}(t)-Q_{f}^{g}(t)\right),$$

(18) $$P_{i}{\left(t\right)}^{2}+Q_{i}{\left(t\right)}^{2}\leq S_{i}(t),$$

where $P_{f}^{d}(t)$ and $Q_{f}^{d}(t)$ are the real and reactive inelastic demand at feeder *f* at time *t*, respectively, and *S*_*i*_(*t*) is the capacity of bank *i* at time *t*.

#### Reserve Commitment:

We consider an ancillary service market of operating reserves [37] in which provision of a certain amount of capacity to track given power commands for a fixed period is paid at a clearing price. Such a market is characterized by a response time *τ*, which specifies the time slots after the commitment when the reserve must be available, and a commitment time *k*, which specifies the number of time slots that the reserve should be kept available. We use the VB to participate in such a reserve market and the required VB energy to put aside in terms of committed reserve power is calculated as follows.

(19) $$P_{\text{rsrv}}^{c}(t)=\sum_{h=\text{max}\{(t-\tau )-k+1,1\}}^{\left(t-\tau \right)}P_{\text{rsrv}}(h),$$

(20) $$P_{\text{rsrv}}^{min}(t)\leq P_{\text{rsrv}}^{c}(t)\leq P_{\text{rsrv}}^{\text{max}}(t),$$

(21) $$B_{\text{rsrv}}(t)=\delta_{t}\sum_{l=\text{max}\{(t-\tau )-k+1,1\}}^{\left(t-\tau \right)}(l-(t-\tau )+k)P_{\text{rsrv}}(l),$$

where $P_{\text{rsrv}}^{c}(t)$ is the cumulative reserve power, and *B*_rsrv_(*t*) is the minimum amount of VB energy required to meet the already committed reserves. To fulfill commitments, the total VB energy has to be maintained above the minimum amount:

(22) $$B_{\text{rsrv}}(t)\leq \sum_{f}B_{f}(t).$$

### 4.2. GML Power Flow Model

The general AC power flow equations,

$$P_{i}=\sum_{k=1}^{\left|B\right|}V_{k}(t)V_{i}(t)\left(G_{ik}\;\text{cos}\left(\theta_{i}(t)-\theta_{k}(t)\right)+B_{ik}\;\text{sin}\left(\theta_{i}(t)-\theta_{k}(t)\right)\right),$$

$$Q_{i}=\sum_{k=1}^{\left|B\right|}V_{k}(t)V_{i}(t)\left(G_{ik}\;\text{sin}\left(\theta_{i}(t)-\theta_{k}(t)\right)-B_{ik}\;\text{cos}\left(\theta_{i}(t)-\theta_{k}(t)\right)\right),$$

are adopted into the GML with a first-order linearization for computational tractability:

(23) $$-\left(P_{i}(t)-P_{i}^{*}(t)\right)=\sum_{k=1}^{\left|B\right|}[V_{k}^{*}(t)\left(G_{ik}\;\text{cos}\left(\theta_{i}^{*}(t)-\theta_{k}^{*}(t)\right)+B_{ik}\;\text{sin}\left(\theta_{i}^{*}(t)-\theta_{k}^{*}(t)\right)\right)\left(V_{i}(t)-V_{i}^{*}(t)\right)\;+V_{i}^{*}(t)\left(G_{ik}\;\text{cos}\left(\theta_{i}^{*}(t)-\theta_{k}^{*}(t)\right)+B_{ik}\;\text{sin}\left(\theta_{i}^{*}(t)-\theta_{k}^{*}(t)\right)\right)\left(V_{k}(t)-V_{k}^{*}(t)\right)\;+V_{i}^{*}(t)V_{k}^{*}(t)\left(-G_{ik}\;\text{sin}\left(\theta_{i}^{*}(t)-\theta_{k}^{*}(t)\right)+B_{ik}\;\text{cos}\left(\theta_{i}^{*}(t)-\theta_{k}^{*}(t)\right)\right)\left(\theta_{i}(t)-\theta_{i}^{*}(t)\right)\;+V_{i}^{*}(t)V_{k}^{*}(t)\left(G_{ik}\;\text{sin}\left(\theta_{i}^{*}(t)-\theta_{k}^{*}(t)\right)-B_{ik}\;\text{cos}\left(\theta_{i}^{*}(t)-\theta_{k}^{*}(t)\right)\right)\left(\theta_{k}(t)-\theta_{k}^{*}(t)\right)],$$

(24) $$-\left(Q_{i}(t)-Q_{i}^{*}(t)\right)=\sum_{k=1}^{\left|B\right|}[V_{k}^{*}(t)\left(G_{ik}\;\text{sin}\left(\theta_{i}^{*}(t)-\theta_{k}^{*}(t)\right)-B_{ik}\;\text{cos}\left(\theta_{i}^{*}(t)-\theta_{k}^{*}(t)\right)\right)\left(V_{i}(t)-V_{i}^{*}(t)\right)\;+V_{i}^{*}(t)\left(G_{ik}\;\text{sin}\left(\theta_{i}^{*}(t)-\theta_{k}^{*}(t)\right)-B_{ik}\;\text{cos}\left(\theta_{i}^{*}(t)-\theta_{k}^{*}(t)\right)\right)\left(V_{k}(t)-V_{k}^{*}(t)\right)\;+V_{i}^{*}(t)V_{k}^{*}(t)\left(G_{ik}\;\text{cos}\left(\theta_{i}^{*}(t)-\theta_{k}^{*}(t)\right)+B_{ik}\;\text{sin}\left(\theta_{i}^{*}(t)-\theta_{k}^{*}(t)\right)\right)\left(\theta_{i}(t)-\theta_{i}^{*}(t)\right)\;+V_{i}^{*}(t)V_{k}^{*}(t)\left(-G_{ik}\;\text{cos}\left(\theta_{i}^{*}(t)-\theta_{k}^{*}(t)\right)-B_{ik}\;\text{sin}\left(\theta_{i}^{*}(t)-\theta_{k}^{*}(t)\right)\right)\left(\theta_{k}(t)-\theta_{k}^{*}(t)\right)],$$

where (*P*_*i*_(*t*), *Q*_*i*_(*t*), *V*_*i*_(*t*), *θ*_*i*_(*t*)) is the set of power flow variables at bank *i* at time *t*, representing respectively the net real power withdrawal, net reactive power withdrawal, voltage magnitude, and phase angle. Accordingly, $\left(P_{i}^{*}(t),Q_{i}^{*}(t),V_{i}^{*}(t),\theta_{i}^{*}(t)\right)$ is a set of power flow setpoints at bank *i* at time *t*, which we shall explain later. *G*_*ik*_ and *B*_*ik*_ are conductance and susceptance between bank *i* and bank *k*, respectively. In particular, we assume that slack bus 0 represents the T&D interface between the TSO and DSO, where the DSO procures power *P*_0_(*t*) and *Q*_0_(*t*) from the wholesale real-time energy market.

### 4.3. GML Formulation and Implementation

At the real-time five-minute timescale, we first propose a scenario-based approach to account for the uncertainty in real-time and reserve prices. Consider a finite set of scenarios S of these price sequences extracted from historical data. For a predetermined sequence range from *t*_1_ to *t*_*f*_, each scenario s∈S is given by

$$s≔\left\{\left(\pi_{s},\lambda_{s}^{rt}(t),\alpha_{s}(t)\right)|t\in \left\{t_{1},t_{2},\cdots t_{f}\right\}\right\},$$

where *π*_*s*_ is the corresponding probability of occurrence with $\sum_{s\in S}\pi_{s}=1$, and $\lambda_{s}^{rt}(t)$ and *α*_*s*_(*t*) are the predicted real-time and reserve prices, respectively.

A receding horizon implementation framework is further put forward to compute the optimal control trajectory. We set a moving prediction window from *t*_*i*_ to *t*_*i*_ + *T* − 1 with all the scenarios in S accounted for. At each time *t* ∈ {*t*_*i*_, ⋯, *t*_*i*_ + *T* − 1}, the decision variables corresponding to scenario s∈S include

$$U_{S}(t)≔\left\{\begin{array}{ll} \left(P_{f,s}^{g}(t),Q_{f,s}^{g}(t),B_{f,s}(t),R_{f,s}(t)\right), & \forall f\in F,\\ \left(P_{i,s}(t),Q_{i,s}(t),v_{i,s}(t)\right), & \forall i\in B,\\ \left(P_{(i,k),s}(t),Q_{(i,k),s}(t),l_{(i,k),s}(t)\right), & \forall (i,k)\in L,\\ \left(P_{\text{rsrv},s}^{c}(t),P_{\text{rsrv},s}(t),B_{\text{rsrv},s}(t)\right) & \end{array}\right\},$$

and the optimization problem to be repeatedly solved is

(25) $$\text{min}\;\sum_{s\in S}\pi_{s}\left[\sum_{t=t_{i}}^{t_{i}+T-1}\delta_{t}\left(\lambda_{s}^{\text{rt}}(t)\left(P_{0,s}(t)-P^{\text{da}}(t)\right)+\lambda^{\text{da}}(t)P^{\text{da}}(t)-\alpha_{s}(t)P_{\text{rsrv},s}(t)+\sum_{f}f_{f,t}\left(P_{f,s}^{g}(t)\right)\right)\right]$$

subject to

(26) $$\text{Secondary}\;\text{feeder}\;\text{constrains}:(10)–(15),\;\forall s\in S\;\text{Bank}\;\text{constraints}:(16)–(18),\;\forall s\in S\;\text{Ancillary}\;\text{service}\;\text{constraints}:(19)–(22),\;\forall s\in S\;\text{Linearized}\;\text{Power}\;\text{flow}\;\text{model}:(23)–(24),\;\forall s\in S\;\text{Scenario}\;\text{coupling}\;\text{constrains}:U_{s}\left(t_{i}\right)=U\left(t_{i}\right),\;\forall s\in S$$

where *P*^da^(*t*) and *λ*^da^ are day-ahead commitment and energy price, respectively. *P*_0,*s*_(*t*) is the net demand acquired in the real-time market at time *t* in scenario *s*. The constraint (26) enforces that the first-slot decision variables *U*(*t*_*i*_) to be implemented immediately are scenario-invariant that couple all scenarios. The term $f_{f,t}\left(P_{f}^{g}\right)$ in (25) captures the solar curtailment cost associated with feeder *f* at time *t* and is explicitly given by

(27) $$f_{f,t}\left(P_{f}^{g}(t)\right)=\beta_{f}\left(P_{f}^{g}(t)-P_{f}^{\text{max}}(t)\right),$$

where $P_{f}^{\text{max}}(t)-P_{f}^{g}(t)$ measures solar curtailment and the constant coefficient *β*_*f*_ < 0 represents its unit cost. We solve for the full trajectory *U*_*s*_(*t*), *t* ∈ [*t*_*i*_, *t*_*i*_ + *T*], but only implement the control action at each current time step *t*_*i*_. The prediction window is then shifted to [*t*_*i*_ + 1, ⋯, *t*_*i*_ + *T*] and repeat the process. Such an iterative solution for the optimization problem mitigates uncertainty through the latest updates of information at each time step.

**Figure 3.**
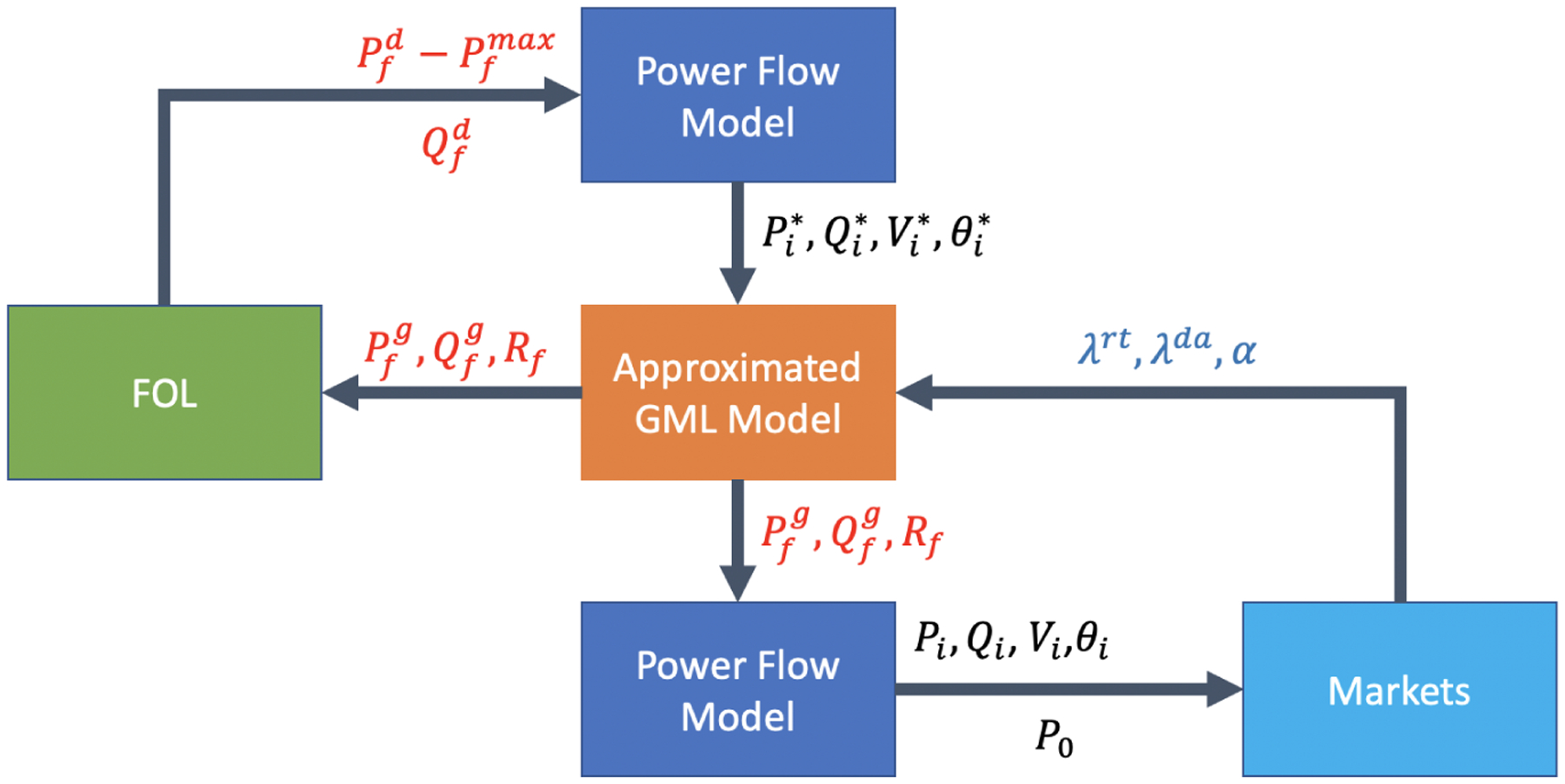
Schematic of the three-layer tuning system.

To compensate for the accuracy loss of the linearized power flows, we introduce the following three-layer tuning mechanism, as depicted in Figure 3. The first and third layers are both power flow layers, which resort to commercial power flow solvers, such as PowerModels.jl [38]. In our first layer, we assume that the demand is given as estimated, the solar generation is provided at its estimate maximum, and the virtual storage remains idle. We then attain the power flow setpoints $\left(P_{i}^{*},Q_{i}^{*},V_{i}^{*},\theta_{i}^{*}\right)$ for ∀i∈B with PowerModels.jl, which are passed to the second layer, the approximate GML model layer. The approximate GML model layer solves for the optimal feeder-level solar and VB scheduling. Note that the resulting power flows are at best approximate, and need to be tuned in the third power flow layer. Through the third layer, accurate power flow is guaranteed based on the solution from PowerModels.jl, given the feeder-level scheduling determined from the second layer.

### 4.4. Peak-Shaving Mode

Peak demand charges constitute a significant portion of the total expenditure for utilities. We therefore include a peak-shaving mode for the real-time GML that takes into account this cost and strikes a trade-off between daily operational cost and peaking demand charge. This mode is expected to be operated only on days where a peak demand is expected.

In particular, we penalize the system for the peak net procurement from the transmission-level market:

(28) $$\text{min}\;\gamma \;\text{max}\;\left\{P_{0,s}(t)\right\}+\sum_{s\in S}\pi_{s}\left[\sum_{t=t_{i}}^{t_{i}+T-1}\delta_{t}\left(\lambda_{s}^{\text{rt}}(t)\left(P_{0,s}(t)-P^{\text{da}}(t)\right)+\lambda^{\text{da}}(t)P^{\text{da}}(t)-\alpha_{s}(t)P_{\text{rsrv},s}(t)+\sum_{f}f_{f,t}\left(P_{f,s}^{g}(t)\right)\right)\right]$$

subject to

Secondaryfeederconstrains:(10)–(15),∀s∈SBankconstraints:(16)–(18),∀s∈SAncillaryserviceconstraints:(19)–(22),∀s∈SLinearizedPowerflowmodel:(23)–(24),∀s∈SScenariocouplingconstrains:(26),∀s∈S

where *γ* is the given peak demand price, commonly way larger than energy clearing prices. The first term in the objective function (28) represents the one-time peak demand charge, and the whole objective function trades off between the GML operational cost and the peak demand charge to achieve a total minimum.

### 4.5. Illustration of GML

We now provide a set of numerical results of a sub-network of New York Independent System Operator (NYISO). The solar generation and demand profiles are obtained from the utility for a day in August 2016. We adopt the price trajectory of August 2019, and consider the following three scenarios in our illustration:

- Scenario #1: This *baseline* scenario assumes that no VB is available, i.e., $B_{f}^{max}(t)=0$, ∀*f*, *t*, and that all solar runs at full capacity, i.e., $P_{f}^{g}(t)=P_{f}^{max}(t)$, ∀*t*, for both the day-ahead and real-time markets.
- Scenario #2: In this *GML* scenario, the GML has the ability to curtail the solar usage and charge/discharge the VB.
- Scenario #3: In this *GML*+*peak-shaving* scenario, the peak-shaving mode is implemented, and the unit price for peak demand charge is set to be *γ* = 10, 000 $/MW.

In Scenarios #2 and #3, we evaluate the economic impact of the VB size. Two sets of VBs are tested: (1) aggregated energy capacity 187.5 MWh and power rating (maximum charging or discharging rate) 75 MW; (2) aggregated energy capacity 375 MWh and power rating 150 MW. The detailed cost comparison is summarized in Table 1. In particular, the day-ahead and real-time costs are both calculated based on net procurement from the transmission-level markets, i.e., $\sum_{t=1}^{T}\lambda^{\text{da}}(t)P_{0}^{\text{da}}(t)\delta_{t}^{\text{da}}$ and $\sum_{t=1}^{T}\lambda^{\text{rt}}(t)\left(P_{0}-P_{0}^{\text{da}}(t)\right)(t)\delta_{t}$. The solar curtailment cost is adopted from (27), and the peak demand charge is explicitly *γ* · max_*t*_ {*P*_0_(*t*)}.

**Figure 4.**
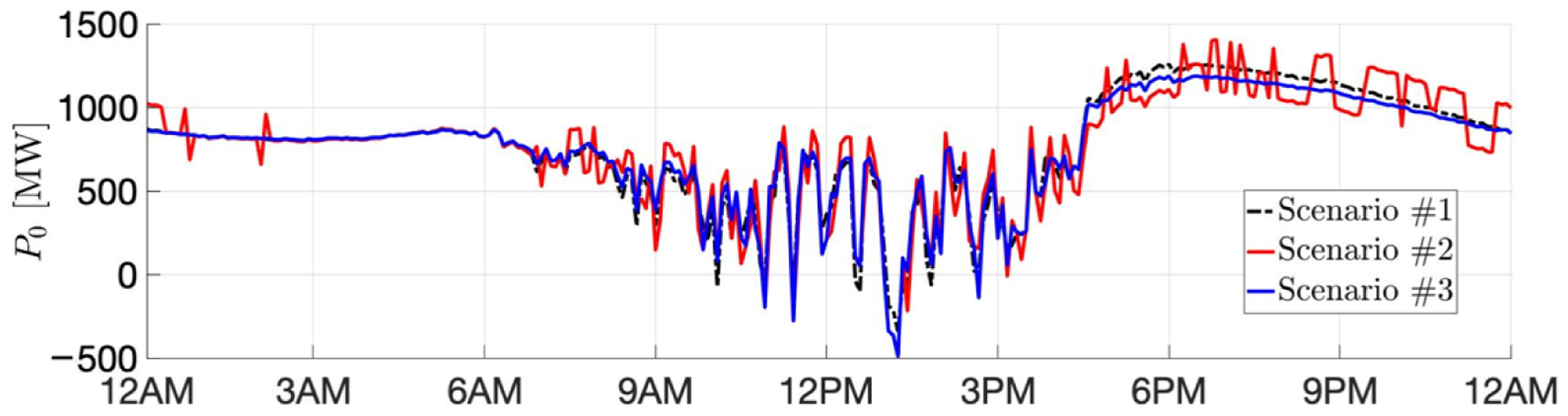
The net procurement *P*_0_ for all three scenarios.

**Figure 5.**
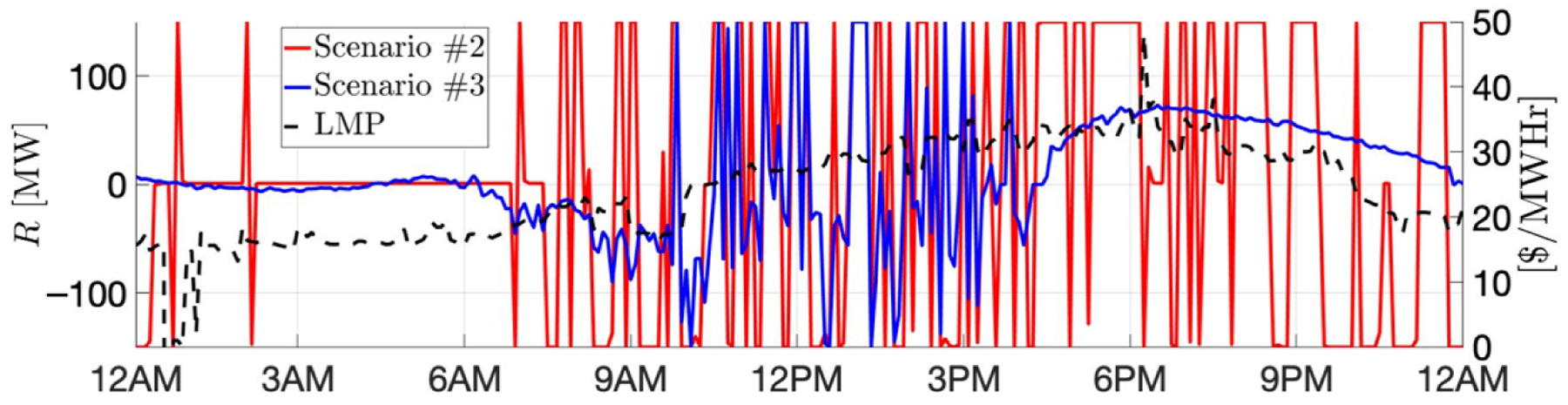
The aggregate virtual battery (VB) discharge with the real-time price. When the price is high, the VB tends to discharge to avoid a high real-time cost, e.g., 18:15.

As we expect, the baseline scenario incurs the highest cost. Given the same VB specifications, the GML scenario reduces the most real-time cost, yet unfortunately creates the highest peak. The lowest peak is guaranteed in Scenario #3. Besides, our results conforms with the intuition that a larger VB leads more savings. Figures 4 and 5 depict the net procurement from the transmission market and the VBs’ aggregate behavior, respectively, for test with VB aggregated energy capacity 375 MWh and power rating 150 MW. We can observe that in Scenario #2, the VB is more active between 17:00 to 20:00, trying to arbitrage across the two-stage markets. However, in Scenario #3, the peak-shaving mode always minimizes the peak net procurement, which tends to flatten the net demand curve.

We further investigate the economic efficiency of these two sets of VBs. The economic efficiency is quantified by the per-unit savings for VB capacity and power rating with respect to the baseline-scenario cost. These results are listed in Table 2, which show that although larger VBs yield more savings, the marginal benefits of VB capacity and power rating decrease.

## 5. Feeder Operational Layer (FOL)

The role of the FOL is to optimally dispatch VBs (i.e., a feeder’s flexibility) to track the GML’s power reference signals at each distribution substation or head node while accounting for the unbalanced AC network physics and the uncertainty inherent to solar PV generation. Figure 1 shows how the FOL interacts with the other layers.

Some key technical challenges associated with the FOL include: (a) the non-convexity of the unbalanced AC power flow equations; (b) the presence of mechanical and continuous controllable resources that result in a mixed integer formulation; (c) the temporal coupling introduced due to the VBs’ energy dynamics; and (d) the need for robustness due to the uncertainty in solar PV generation. To overcome the challenge of dispatching both slower mechanically-actuated grid assets, such as LTCs or capacitor banks, and flexible and responsive VBs, we decompose the FOL into a slow outer loop and a fast inner loop. The different timescales of operation allow for mechanical and flexible resources to serve different purposes. Specifically, the outer loop of the FOL employs a simplified power flow model and focuses on dispatching the discrete mechanical assets to maximize voltage margins while accounting for expected solar PV generation. The inner loop then adopts the outer loop’s mechanical asset dispatch and employs a full unbalanced AC power flow to optimize the dispatch of VBs to track the GML’s reference signal. Details on the outer loop formulation for various mechanical assets are presented in detail in [28,39]. In this manuscript, the focus will be on inner loop and optimal dispatch of VBs and PV inverters to track the GML’s reference signal at the head node.

**Figure 6.**
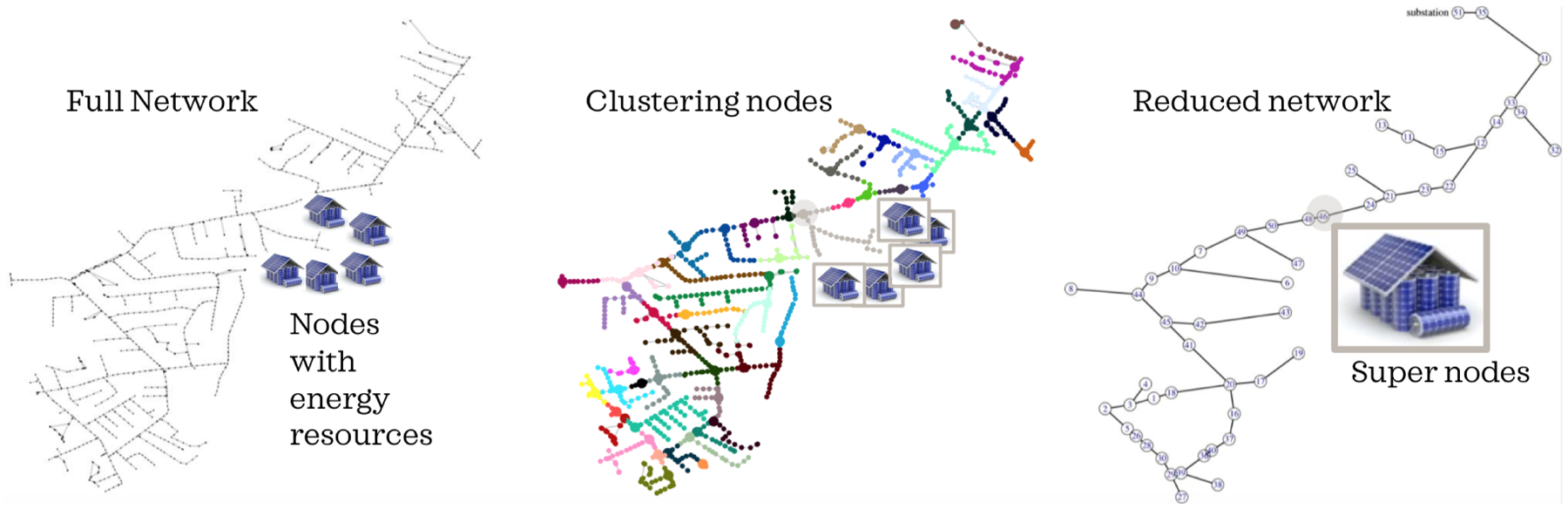
Process for network reduction of electrical circuits by partitioning the network into clusters of similar nodes with the same color. The largest nodes in each cluster is the designated super-node.

**Remark 1** (Large-scale networks). *Nonlinear, unbalanced AC power flow equations beget optimization algorithms that do not scale well as the network size increases. To ensure scalability for the proposed OPF algorithms in the FOL, we approximate the full network as illustrated in* Figure 6 *using Kron reduction* [40]*. This is achieved by systematically creating clusters of electrically similar and proximal nodes using voltage sensitivities to current injections* [41]*. For each cluster, we then designate a “super-node” from which we can employ a 3-phase Kron reduction* [42]*. Within each cluster, solar PV and demand are then aggregated up to the corresponding super-node. In addition, the STL then coordinates all devices within the same cluster, which represents a VB based on less than 200 flexible DERs per super-node in this manuscript. While the reduced network does represent a physically meaningful approximation of the full network and we find maximum intra-cluster voltage magnitude errors of less than 0.015 pu across a wide range of operations, work is ongoing to study optimal network reductions and the role of the intra-cluster networks in constraining VB and feeder flexibility. For now, we utilize the intra-cluster error bounds to tighten voltage bounds and ensure an OPF formulation that is robust to model mismatch*.

### 5.1. FOL Multi-Period Formulation

The physics that define the unbalanced AC power flows in (1)–(5) are used to formulate the FOL’s optimal reference-tracking VB dispatch problem. However, these equations are non-linear due to (3) and lead to a non-convex optimization problem. To achieve an efficient formulation, we employ a second order conic (SOC) relaxation of (3), which is based on [43] and detailed in [27].

The set of decisions variables over which we optimize in the FOL are $\left\{P_{n}^{\text{b}}(t),S_{n}^{\text{S}}(t)\right\}$ for each node n∈N and time *t*. These decision variables affect the dependent variables *W*_*n*_(*t*), *S*_*l*_(*t*), *I*_*l*_(*t*), $S_{n}^{\text{net}}(t)$, *B*_*n*_(*t*) at each node *n* and branch *l*. Finally, the FOL leverages the following data as constant parameters: *Z*_*l*_, $S_{n}^{\text{L}}(t)$, *S*_max,*l*_, *V*_min,*n*_, *V*_max,*n*_, *G*_max,*n*_, *η*_c,*n*_, *η*_d,*n*_, *H*_max,*n*_, *B*_min,*n*_, *B*_max,*n*_, *P*_max,*n*_ for nodes *n* and branches *l*. If we define the FOL’s prediction horizon as T={0,1,…,T−1}, then the problem of dispatching the VBs to optimally track the GML’s head node power reference can be formulated as:

(29) $$\underset{x}{\text{min}}f_{1}(x)$$

(30) subjectto:(1),(2),(4),(5)

(31) $$B_{n}(t+1)=B_{n}(t)-P_{n}^{\text{b}}(t)\Delta t\;\forall n\in G$$

(32) $${\left\|\frac{2W_{n}(t)(i,j)}{W_{n}(t)(i,i)-W_{n}(t)(j,j)}\right\|}_{2}\leq W_{n}(t)(i,i)+W_{n}(t)(j,j)$$

(33) $${\left\|\frac{2I_{l}(t)(i,j)}{I_{l}(t)(i,i)-I_{l}(t)(j,j)}\right\|}_{2}\leq I_{l}(t)(i,i)+I_{l}(t)(j,j)$$

(34) $${\left\|\frac{2S_{l}(t)(i,j)}{W_{n}(t)(i,i)-I_{l}(t)(j,j)}\right\|}_{2}\leq W_{n}(t)(i,i)+I_{l}(t)(j,j)$$

(35) $$\left|\text{diag}\left(S_{l}(t)\right)\right|\leq S_{\text{max},l}\;\forall l\in L$$

(36) $$V_{\text{min},n}^{2}\leq \text{diag}\left(W_{n}(t)\right)\leq V_{\text{max},n}^{2}\;\forall n\in N$$

(37) $$\left|S_{n}^{\text{S}}(t)\right|\leq G_{\text{max},n}\;\forall n\in G$$

(38) $${\left(P_{n}^{\text{b}}(t)\right)}^{2}+{\left(q_{n}^{\text{b}}(t)\right)}^{2}\leq H_{\text{max},n}^{2}\;\forall n\in G$$

(39) $$B_{\text{min},n}\leq B_{n}(t)\leq B_{\text{max},n}\;\forall n\in G$$

(40) $$P_{\text{min},n}\leq P_{n}^{\text{b}}(t)\leq P_{\text{max},n}\;\forall n\in G$$

for t∈T, where the reference-tracking objective function in (29) is given by:

$$f_{1}(x)≔\sum_{t\in T}\left({\left(L_{1}(t)+P^{\text{GML}}(t)-\sum_{n\in N}\text{real}\left\{S_{n}^{\text{net}}(t)\right\}\right)}^{2}+\alpha {\left(R_{f}(t)-\sum_{n\in N}P_{n}^{\text{b}}(t)\right)}^{2}+\beta {\left(P_{f}^{\text{g}}(t)-\sum_{n\in N}\text{real}\left\{S_{n}^{\text{S}}(t)\right\}\right)}^{2}+\gamma {\left(Q_{f}^{\text{g}}(t)-\sum_{n\in N}\text{imag}\left\{S_{n}^{\text{S}}(t)\right\}\right)}^{2}+\epsilon \sum_{l\in L}1^{⊤}\text{diag}\left(R_{l}\circ I_{l}(t)\right)\right).$$

The first term in *f*_1_(*x*) represents the tracking of the feeder head-node power signal *P*^GML^ with $L_{1}(t)=L_{0}(t)+\sum_{n\in N}\zeta_{n}\Delta p_{n}(t)$ being a first-order approximations of the total feeder line losses, *L*_0_(*t*) is the loss estimated for the operating point at time *t*, and *ζ*_*n*_Δ*p*_*n*_(*t*) represents the change in total feeder losses due to the change in active power injection at node *n*. The factors *ζ*_*n*_ represent the sensitivity in feeder losses due to changes in active power injections and are similar to the power transfer distribution factors (PTDFs) that are often used in transmission system analysis [44]. The second term enforces tracking of the GML VB reference setpoint *R*_*f*_ for feeder *f*. The third term tracks the GML solar PV reference $P_{f}^{\text{g}}$, the fourth term tracks the GML reactive power reference $Q_{f}^{\text{g}}$ and the final term additionally minimizes feeder losses. Inequality (35) bounds the line power flow below apparent power limit $S_{\text{max},l}\in {\mathbb{R}}^{\left|\phi \right|}$, while (36) captures the voltage bounds at each node with $V_{\text{min},n}\in {\mathbb{R}}^{\left|\phi \right|}$ and $V_{\text{max},n}\in {\mathbb{R}}^{\left|\phi \right|}$ as the lower and upper voltage limits, respectively, and inequality (37) bounds the apparent power of the solar inverter. Inequalities (38)–(40) define bounds on VB apparent power, state of charge (SoC), and active power dispatch, respectively. Specifically, $H_{\text{max},n}\in {\mathbb{R}}^{\left|\phi \right|}$ defines the apparent power limit of the corresponding VB’s complex power injection and *B*_min,*n*_, $B_{\text{max},n}\in {\mathbb{R}}^{\left|\phi \right|}$ and *P*_min,*n*_, $P_{\text{max},n}\in {\mathbb{R}}^{\left|\phi \right|}$ are the VB’s lower and upper energy and power bounds, respectively. The relation between the battery SoC and battery power is given by (31), where Δ*t* is the width of the discrete time steps. In this work, we employ the simplifying assumption that VBs have unity charge/discharge efficiencies, which avoids the technicalities around simultaneous charging and discharging, which is reasonable for VBs as explained in [45] and represents ongoing work [27,46].

Since (32)–(34) are a convex relaxation of the nonlinear (3), the resulting optimal solution may not represent the exact underlying physics (i.e., the relaxation may have a non-zero duality gap). This means that any optimal VB dispatch could for example employ “fictitious” losses to improve tracking or voltage magnitudes. To ensure a physical and AC-admissible optimal dispatch, we augment the FOL with another OPF layer that is based on a nonlinear programming (NLP) formulation of the OPF problem that is initialized with the SOCP’s solution over the prediction horizon. Since the SOCP formulation already accounts for the multiple time-steps related to the VB energy dynamics, an NLP formulation can be judiciously designed. This is described next.

#### 5.1.1. Ensuring AC Feasible Optimal Solution

In general, multi-period, non-convex NLP problems scale poorly, but we can utilize the “warm start” provided by the multi-period SOCP problem. Specifically, we leverage the authors’ prior work in [27], where the solution obtained from a similar multi-period SOCP problem is passed to a multi-period, non-convex NLP formulation that fixes the VB’s active-power set-points to match that of the SOCP’s optimal solution over the entire prediction horizon. This effectively keeps the VB’s energy trajectory constant and enables a decomposition of the multi-period NLP formulation into *T* decoupled non-convex NLPs. Thus, after solving the multi-period SOCP, we can ensure an AC-feasible optimal VB dispatch by efficiently solving the *T* independent NLPs in parallel.

Thus, for all t∈T, the NLP formulation is given by:

(41) $$\underset{x}{\text{min}}f_{2}(x(t))$$

(42) subjectto:(1)–(5),(35)–(38)

(43) $$P_{n}^{\text{b}}(t)=P_{n}^{\text{b}*}\;\forall \text{n}\in G$$

where objective $f_{2}(x(t))≔{\left(Q_{f}^{\text{g}}(t)-\sum_{n\in N}\text{imag}\left\{S_{n}^{\text{S}}(t)\right\}\right)}^{2}+\epsilon \sum_{l=1}^{L}1^{T}\text{diag}\left(R_{l}\circ I_{l}(t)\right)$ only corrects the controllable reactive power set-points and $P_{n}^{\text{b}*}\in R^{\left|\phi \right|}$ is the optimal active power injection of the VB at node *n* obtained from the SOCP. The time-decoupled NLP is then solved for the entire prediction horizon in parallel to obtain an AC-feasible dispatch of flexible resources in unbalanced feeders, including the VBs and solar PV inverters. Thus, the coupled SOCP-NLP optimization framework, represents a scalable approach for the FOL to optimize resources in realistic unbalanced feeders.

### 5.2. Robust FOL Formulation

Since we are employing predictive optimization in the FOL, we need to consider the inherent uncertainty in solar PV and demand predictions and render the FOL formulation robust against the intra-hour forecast errors. A chance-constraint-based optimization is employed herein to achieve a robust dispatch of flexible resources. Thus, we need to characterize the uncertainties involved to determine an intuitive probabilistic security level [47].

#### 5.2.1. Nature of Uncertainty in Solar PV Forecasts

**Figure 7.**
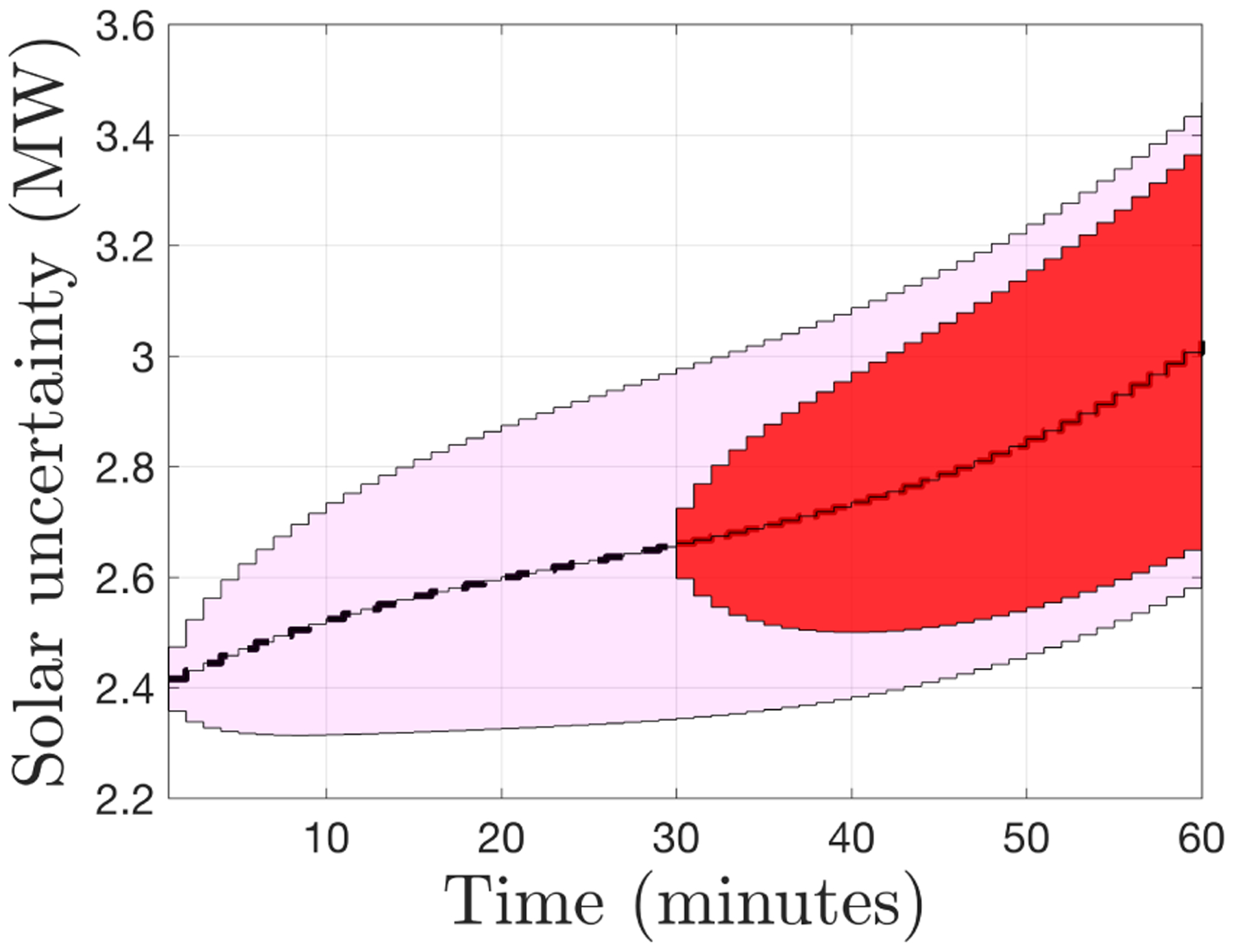
Illustrating the effect of the intra-hour forecast error model for solar PV forecast over the prediction horizon. The thick dashed line (**- -**) represent the expected solar PV generation. The forecasts provide a 60-minute preview window and are updated every 30 m.

To illustrate the uncertainty in intra-hourly solar PV forecasts, we consider solar PV forecast errors based on relative root-mean-square error (rRMSE) as presented in [29]. In this model, as forecasts predict further ahead, the rRMSE increases logarithmically. For example, the rRMSE is about 15% looking 20 min ahead and about 20% looking 60 min ahead. Two realizations of this solar PV forecast error model are shown in Figure 7, where the forecast looks ahead one hour and is updated every 30 min. The colored area represents uncertainty around the expected solar PV generation of each forecast. The presented forecast error model is meant to be representative of today’s state-of-the-science in intra-hourly (very short-term) solar PV forecasts [48,49].

In the FOL, we have assumed that these minutely solar PV (and similar demand) forecasts are available to the DSO over the entire prediction horizon and are updated every 30 min [50]. In this FOL, we assume that forecast errors are uniformly distributed within the range provided (i.e., each point in the range is equally likely), which means the we have a unimodal distribution and can employ the recently-developed unimodal Chebyshev approximation within the framework of chance constraints [45].

#### 5.2.2. Chance-Constraints

The solutions obtained from the time-decoupled NLP problems are used as operating points about which we can linearize the unbalanced AC power flow model at each time-step. Based on the obtained linear models, over the entire prediction horizon, the uncertainty in demand and solar PV along with sensitivity factors, similar to those in [29], are used to systematically tighten the voltage and power flow constraints and robustly solve the AC OPF at the next instant.

For example, consider optimization variable *Y* (e.g., voltage magnitude), which has sensitivity factor Γ_*Y*Ω_ with respect to the random variable Ω (e.g., predicted solar PV generation). Using the sensitivity factor allow us to easily express chance constraints such as $\mathbb{P}\left(Y+\Gamma_{Y\Omega }\Omega \leq Y_{max}\right)\geq 1-\alpha_{\text{Y}}$ where 1 − *α*_Y_ represents the acceptable probability level with *α*_Y_ > 0. Clearly, as *α*_Y_ → 0, the set of feasible actions gets smaller (i.e., solution becomes more conservative). In this form, we can directly apply the analytical reformulation of the chance constraint to engender a robust, but deterministic formulation that achieves robustness by appropriately tightening voltage and line flow bounds [51]. The tightened bounds ensure that the optimal solution is robust against desired uncertainty levels (in the linearized model). However, tightening bounds can lead to infeasibility, which is overcome by penalizing slack variables, e.g., $Y_{\text{v}}^{+}$ and $Y_{\text{v}}^{-}$, in the objective function. Based on the above outlined approach, we can now formulate a robust version of the deterministic, multi-period SOCP optimization problem as follows:

(44) $$\underset{x}{\text{min}}f_{1}(x)+\eta \sum_{t\in T}\sum_{n=1}^{N}1^{T}\left(V_{\text{v},n}^{+}(t)+V_{\text{v},n}^{-}(t)\right)$$

(45) subjectto:(1),(2),(4),(5)

(46) $$B_{n}(t+1)=B_{n}(t)-P_{n}^{\text{b}}(t)\Delta t\;\forall n\in G,$$

(47) $${\left\|\frac{2W_{n}(t)(i,j)}{W_{n}(t)(i,i)-W_{n}(t)(j,j)}\right\|}_{2}\leq W_{n}(t)(i,i)+W_{n}(t)(j,j),$$

(48) $${\left\|\frac{2I_{l}(t)(i,j)}{I_{l}(t)(i,i)-I_{l}(t)(j,j)}\right\|}_{2}\leq I_{l}(t)(i,i)+I_{l}(t)(j,j),$$

(49) $${\left\|\frac{2S_{l}(t)(i,j)}{W_{n}(t)(i,i)-I_{l}(t)(j,j)}\right\|}_{2}\leq W_{n}(t)(i,i)+I_{l}(t)(j,j),$$

(50) $$\left|\text{diag}\left(S_{l}(t)\right)\right|\leq {\bar{L}}_{\text{b},l}\left(t,\alpha_{\text{L}},\Sigma \right)\;\forall l\in L,$$

(51) $${\underline{V}}_{\text{b},n}\left(t,\alpha_{\text{v}},\Sigma \right)-V_{\text{v},n}^{-}(t)\leq \text{diag}\left(W_{n}(t)\right)\leq {\bar{V}}_{\text{b},n}\left(t,\alpha_{\text{v}},\Sigma \right)+V_{\text{v},n}^{+}(t)\;\forall n\in N,$$

(52) $$\left|S_{n}^{\text{S}}(t)\right|\leq {\bar{S}}_{\text{b},n}\left(t,\alpha_{\text{s}},\Sigma \right)\;\forall n\in G,$$

(53) $${\left(P_{n}^{\text{b}}(t)\right)}^{2}+{\left(q_{n}^{\text{b}}(t)\right)}^{2}\leq H_{\text{max},n}^{2}\;\forall n\in G,$$

(54) $$B_{\text{min},n}\leq B_{n}(t)\leq B_{\text{max},n}\;\forall n\in G,$$

(55) $$P_{\text{min},n}\leq P_{n}^{\text{b}}(t)\leq P_{\text{max},n}\;\forall n\in G$$

for all t∈T, where $V_{\text{v},n}{\left(t\right)}^{+}\in {\mathbb{R}}^{\left|\phi \right|}$ and $V_{\text{v},n}{\left(t\right)}^{-}\in {\mathbb{R}}^{\left|\phi \right|}$ represent the slack variables that are added to ensure persistent feasibility for the upper and lower voltage bounds, respectively, and with *η* ≫ 1. Inequalities (53)–(55) define bounds on VB apparent power, state of charge (SoC), and active power dispatch, respectively. The relation between the battery SoC and battery power is similarly given by (46). The constraints (47)–(49) are a convex relaxation of the nonlinear (3). The *α*_L_-robust bound for apparent line flows is given by ${\bar{L}}_{\text{b},l}\left(t,\alpha_{\text{L}},\Sigma \right)≔S_{\text{max},l}-\lambda_{\text{L}}\left(\alpha_{\text{L}},\Sigma ,S_{l}{\left(t\right)}^{*}\right)$, while the *α*_v_-robust voltage bounds are given by ${\bar{V}}_{\text{b},n}\left(t,\alpha_{\text{v}},\Sigma \right)≔V_{\text{max},n}^{2}-\lambda_{\text{v}}\left(\alpha_{\text{v}},\Sigma ,W_{n}{\left(t\right)}^{*}\right)$, ${\underline{V}}_{\text{b},n}\left(t,\alpha_{\text{v}},\Sigma \right)≔V_{\text{min},n}^{2}+\lambda_{\text{v}}\left(\alpha_{\text{v}},\Sigma ,W_{n}{\left(t\right)}^{*}\right)$. Similarly, apparent solar inverter power bounds can be made tightened as ${\bar{S}}_{\text{b},n}\left(t,\alpha_{\text{s}},\Sigma \right)≔G_{\text{max},n}-\lambda_{\text{s}}\left(\alpha_{\text{s}},\Sigma ,S_{n}^{\text{S}*}(t)\right)$. Note that the bounds are tightened by entity $\lambda_{\text{Y}}\left(\alpha_{\text{Y}},\Sigma ,Y^{*}\right)≔f_{\text{sff}}^{-1}\left(1-\alpha_{\text{Y}}\right){\left\|\Gamma_{Y\Omega }\Sigma^{1/2}\right\|}_{2}$ which shows that the tightening depends on both the operating point Y* and the so-called *safety factor function* $f_{\text{sff}}^{-1}\left(1-\alpha_{\text{Y}}\right)$, which is defined by the acceptable probability level. The safety-factor function for the unimodal distribution employed herein is an approximation based on the exact numerical solution from [52] and is given by

$$f_{\text{sff}}^{-1}\left(1-\alpha_{\text{Y}}\right)<{\left(\frac{1-\alpha_{\text{Y}}}{e\alpha_{\text{Y}}}\right)}^{1/1.95},$$

where *e* is Euler’s number. As indicated by the strict inequality, this approximation is, in fact, a tight inner approximation of $f_{\text{sff}}^{-1}\left(1-\alpha_{\text{Y}}\right)$, i.e., no less conservative, as detailed in [45].

Similarly, the deterministic, time-decoupled NLP optimization in (41)–(43) that must be solved for each time-step in the prediction horizon are also made robust against forecast errors by tightened voltage and line flow bounds to form the following decoupled robust NLPs for each timestep t∈T:

(56) $$\underset{x}{\text{min}}f_{2}(x(t))+\eta \sum_{n=1}^{N}1^{T}\left(V_{\text{v},n}^{+}(t)+V_{\text{v},n}^{-}(t)\right)$$

(57) subjectto:(1)–(5)

(58) $${\left(P_{n}^{\text{b}*}\right)}^{2}+{\left(q_{n}^{\text{b}}(t)\right)}^{2}\leq H_{\text{max},n}^{2}\;\forall n\in G$$

(59) $$\left|\text{diag}\left(S_{l}(t)\right)\right|\leq {\bar{L}}_{\text{b},l}\left(t,\alpha_{\text{L}},\Sigma \right)\;\forall l\in L$$

(60) $${\underline{V}}_{\text{b},n}\left(t,\alpha_{\text{v}},\Sigma \right)-V_{\text{v},n}^{-}(t)\leq \text{diag}(W_{n}(t)\leq {\bar{V}}_{\text{b},n}\left(t,\alpha_{\text{v}},\Sigma \right)+V_{\text{v},n}^{+}(t)\;\forall n\in N$$

(61) $$\left|S_{n}^{\text{S}}(t)\right|\leq {\bar{S}}_{\text{b},n}\left(t,\alpha_{\text{s}},\Sigma \right)\;\forall n\in G$$

where (1)–(5) represents the nonlinear power flow equations and (58) represents the VB apparent power constraint with $P_{n}^{\text{b}*}\in R^{\left|\phi \right|}$ being the optimal active power injection of the VB at node *n* obtained from the solution of the robust SOCP given in (44)–(55) Thus, the FOL’s inner loop is the combination of the robust, multi-period SOCP formulation in (44)–(55) and the robust, time-decoupled NLPs in (56)–(61).

### 5.3. Illustration of FOL with Solar PV Forecasts

In this section, we illustrate the inner loop of the FOL with a realistic example of an unbalanced distribution feeder. Specifically, simulations are conducted on a 1200-node unbalanced feeder with a voltage base of 7.6 kV that has been Kron-reduced into a reduced, representative distribution feeder with 130 “super nodes” and the head-node (i.e., distribution substation).

The robust SOCP-NLP algorithm is implemented minutely over an hour in a receding-horizon fashion with a prediction and control horizon of ten time-steps. Thus, the SOCP results in an open-loop, optimal VB and PV inverter power dispatch trajectory, which is used by the NLP instances to compute an optimal AC-feasible power dispatch trajectory. The dispatch trajectory is then used to update the sensitivity-based bound tightening process at every time step. The multi-period SOCP is solved using GUROBI [53], whereas the NLPs are solved with IPOPT [54] using library HSL_MA86 [55] and the total compute time for the SOCP-NLP is no worse than 15 seconds to ensure viable methodology. Based on the FOL’s optimal dispatch and the actual demand and solar PV injections, AC load flows are computed with GridLab-D [56].

**Figure 8.**
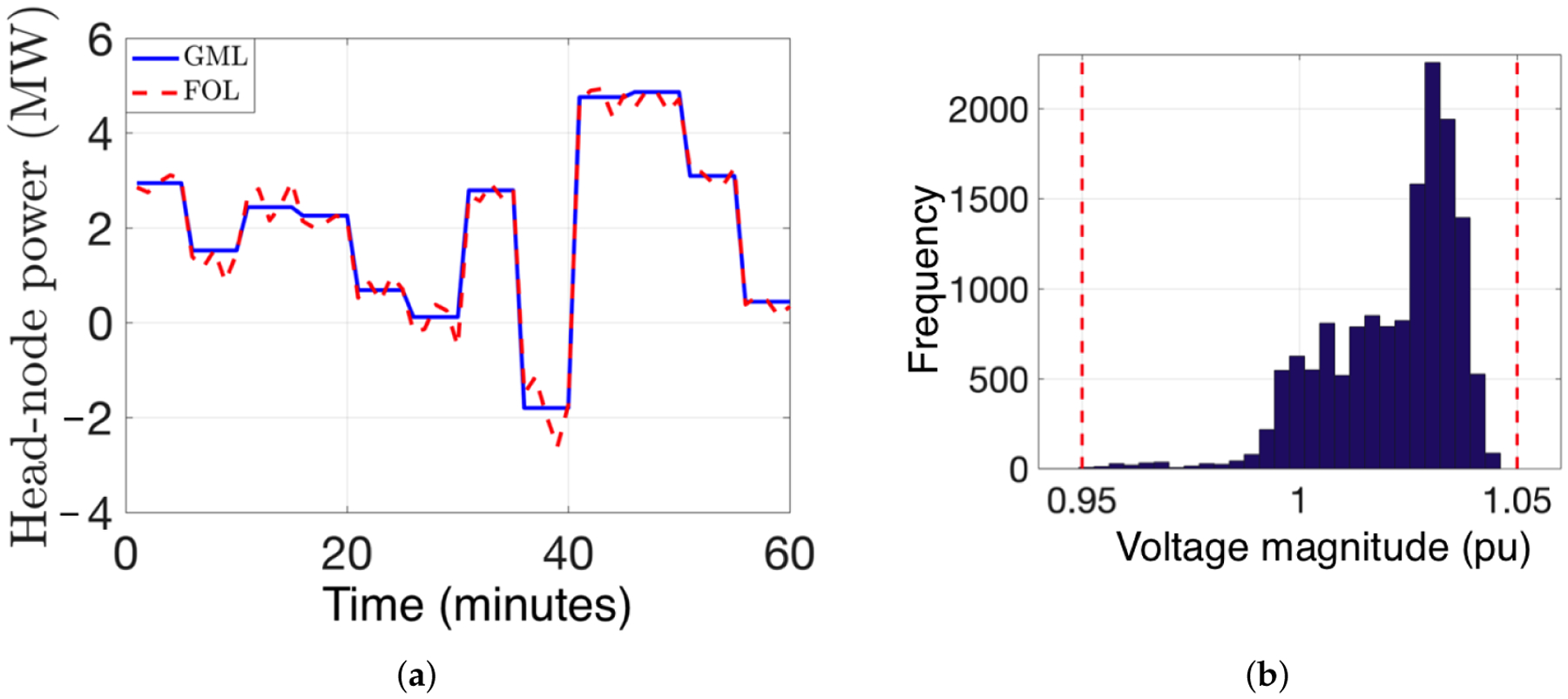
*Left:* (**a**) Tracking of the reference grid market layer (GML) head-node power by the feeder operational layer (FOL) through the control of VBs showing acceptable tracking performance for the period 13:00–14:00 *Right:* (**b**) Histogram of the voltages obtained from the stochastic AC OPF. Clearly, the voltages are within the ANSI limits given by the red dashed vertical lines.

We illustrate, in Figure 8a, the effectiveness of the FOL’s robust inner loop in tracking a GML power reference at the headnode, including reverse flows (when head-node power is negative). Note that the realized voltage magnitudes in Figure 8b are within the ANSI limits of [0.95, 1.05] pu despite forecast errors in solar PV output based on the model illustrated in Figure 7. The robust outcome was achieved with *α*_v_ = 0.05.

## 6. Service Transformer Layer (STL)

The two key tasks of each STL element are: (i) *characterize flexibility of a DER fleet*, i.e., updating and reporting to the FOL every 1 min to 5 min the VB parameters presented in Section 2 for the small fleet of *N* DERs that sit behind each local service transformer or behind a local cluster of service transformers in the case of a super node; and (ii) *DER coordination*, i.e., real-time control of those DERs to track FOL-dispatched set-points for active and reactive power once every 1 s to 4 s. Section 2.3 presents closed-form expressions which allows the STL elements to quickly update the VB flexibility models based on (updated) information regarding the availability and parameters of the individual DERs. In this section, we focus on the real-time DER coordination task executed by the STL elements.

The STL controller performs a real-time optimal dispatch of the DERs (e.g., switching thermostatic loads on/off) to track the power set-points by explicitly accounting for service transformer and DER quality of service constraints, as necessary [25]. In particular, this dispatch of control signals to a group of responsive ACs and WHs is performed via the following optimization scheme which ensures that a set-point trajectory at the service transformer level is tracked with constraints ensuring that end-user comfort specifications are met during the tracking performance:

(62) $$\forall t:\;\underset{\varepsilon >0,{\left\{p_{i}\right\}}_{i=1}^{N}}{\text{minimize}}\;w_{1}\varepsilon +w_{2}\sum_{i=1}^{N}{\left\|T_{i}(t+1)-T_{set,i}\right\|}_{2}^{2}$$

(63) $$\text{subject}\;\text{to}\;\left|P_{set}(t)-\sum_{i=1}^{N}p_{i}\right|\leq \varepsilon ,$$

(64) $$T_{i}(t+1)\in \left[T_{set,i}-\delta T_{i}/2,T_{set,i}+\delta T_{i}/2\right]\;\forall i,$$

(65) $$p_{i}\in \left\{0,P_{i}\right\}\;\forall i,$$

where *w*_1,2_ > 0 are some weights, *ε* > 0 is the allowable tracking error, *P*_*set*_(*t*) is the FOL-dispatched (active) power set-point at time *t*, *P*_*i*_ is the rated power of device *i*, and ${\left\{T_{i}(t+1)\right\}}_{i=1}^{N}$ denote the predicted temperature state variables of the thermostatic loads (room temperature for ACs, water temperature for WHs) obtained from discretized versions of the model Equations (6) and (7) as a function of the decision variables which are the device power consumption values ${\left\{p_{i}\right\}}_{i=1}^{N}$. The temperature set points and dead band temperature limits for the thermostatic loads are given by ${\left\{T_{set,i}\right\}}_{i=1}^{N}$ and ${\left\{\delta T_{i}\right\}}_{i=1}^{N}$. Notice that this optimization problem is essentially a *mixed-integer-problem* (MIP) due to the binary constraints on the optimization variable *p*_*i*_ (on/off status of each device), which we can solve rather efficiently using open-source solvers such as Cbc by COIN-OR [57]. Note, however, that there are other alternatives to solving this problem by relaxing each of the binary constraints and adding appropriate penalty terms in the objective, as described in [25,58]. Ongoing efforts are investigating adding more device-specific requirements, such as cycling constraints, to the problem.

**Figure 9.**
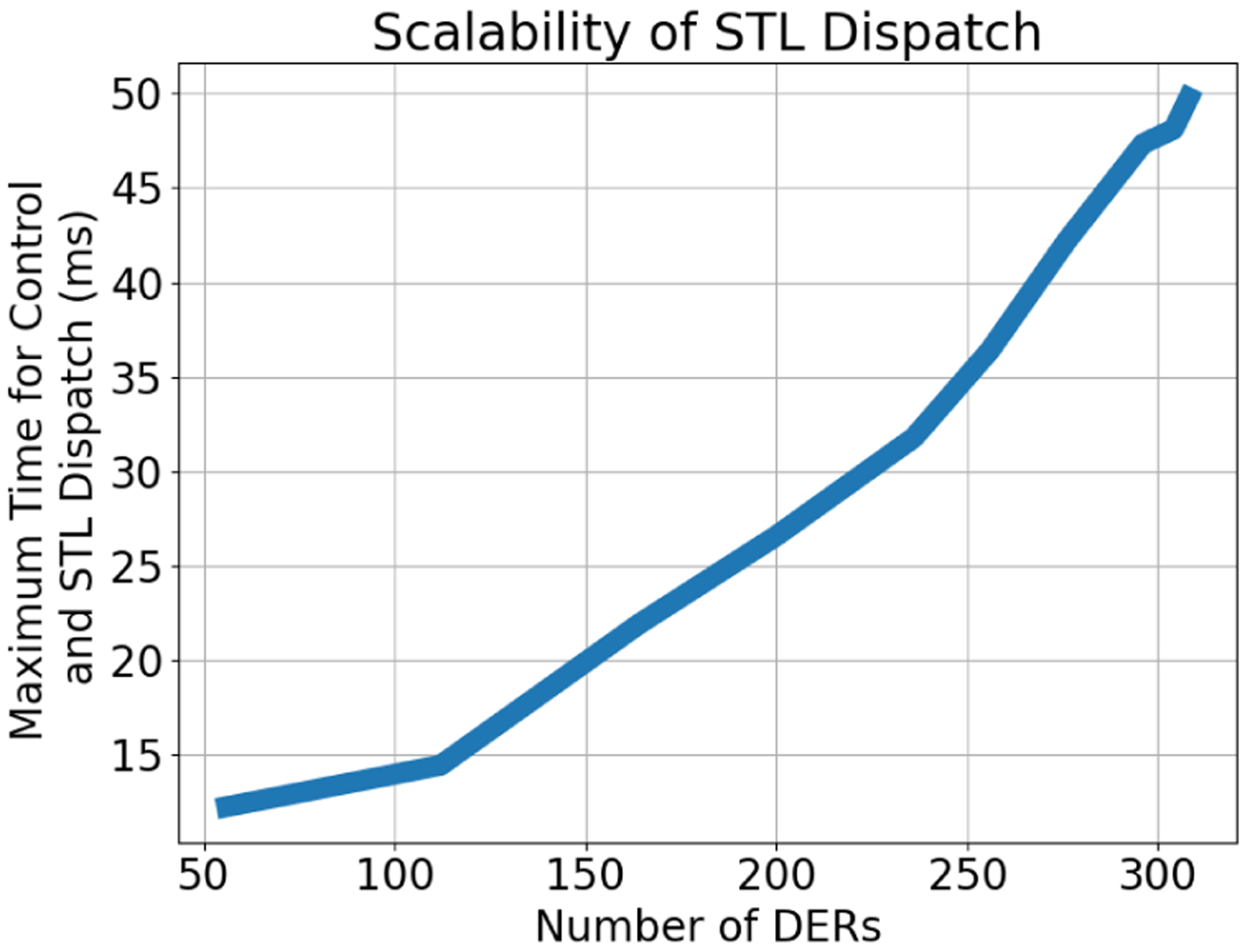
Scalability of real-time control and service transformer layer (STL) coordination of distributed energy resources (DERs).

Figure 9 shows the time required to solve (62)–(65) as a function of the number of DERs. Clearly, the sub-100ms computation times makes the STL suitable for the desired DER dispatch and intra-feeder control. The intra-feeder control scheme updates *P*_set_ based on the feeder’s realtime head-node measurement and the FOL’s tracking error and is described next in Section 7.

## 7. Inter-Layer Communication and Control

The presented three-layer approach in Figure 1 represents a utility-centric scheme for managing a distribution system with millions of actively controlled DERs. Thus, the scheme has access to:

1. The full network data for the FOL’s optimization-based dispatch of VBs.
2. Live SCADA and power flow information from distribution substations.
3. Secure communication infrastructure for corrective inter-feeder and intra-feeder control.

These assumptions are consistent with the ongoing developments to the IEEE 2030 interoperability guides and standards for “inter-domain” and “intra-domain” communication and control in electric power systems [59]. The following subsection outlines one potential information architecture for inter-layer communications to facilitate dispatch and control between the GML and FOL (inter-feeder); corrective control between FOL and STL (intra-feeder); real-time control between STL and its fleet of behind-the-service-transformer DERs.

### 7.1. Communications between Layers

Figure 1 illustrates the notion of layers in the proposed hierarchical DER control scheme. Communication between these layers has so far been implicitly assumed to share common information models and communication protocols. Recall that the inter-feeder controller supports operations at the T&D interfaces of the TSO and DSO by correcting set-points at the MV distribution substations based on total DSO in-feed at an HV transmission or sub-transmission bus. This HV substation can be considered a point of common coupling between all distribution feeders and also receives wholesale market price signals (e.g., $/MWh). That is, we have made the reasonable assumption in this work that there exists an HV TSO substation to which a collection of feeders are electrically coupled and for which the DSO (via the GML) acts as a scheduling coordinator with respect to TSO markets. From a communication point of view, this interface also represents a key assumption about the compositionality of the information models used to represent thousands of individual DERs, the primary and secondary distribution circuit, and MV/LV transformers - that they can be expressed as a single abstract asset connected at the DSO in-feed to the sub-transmission substation. As a corollary, power setpoints transmitted from the TSO’s HV transmission/sub-transmission substation remain consistent when dis-aggregated down to all the MV distribution substations (i.e., head-nodes) for each feeder, which in turn are interpreted by a distribution substation SCADA system [60]. Our implicit assumptions about scalable hierarchical communications are based on the following specific architectural predicates.

Corrective interactions between GML and FOL require effective bi-directional information exchange at the substation SCADA on the order of 5 s. Information includes inputs to the inter-feeder controller and tracking error inputs to the intra-feeder controller. These inputs can be obtained using in-feed and out-feed measurements of power, voltage, and breaker state at a HV-MV distribution substation and downstream MV-LV service transformer interfaces, respectively. The inputs to the intra-feeder controller may be obtained using distribution substation devices called real-time automation controllers (RTACs [61,62]) that have 100 to 500 ms update loops. Importantly, the costs of devices like RTACs have reduced to within the order of magnitude of embedded DER interfaces, making it feasible that each distribution substation is equipped with an RTAC and related communication capabilities.

The intra-feeder controller operates between the FOL’s primary and the STL’s secondary distribution circuits and is responsible for real-time corrective control of the FOL’s dispatched VB power set-points. Real-time corrections can benefit from faster streaming measurements of vector power flows within the primary distribution network and the aggregate power flows at the head-node (MV substation). This real-time capability can be adequately serviced with the use of distribution circuit optimized phasor measurement units [63,64]. Here too, the costs of instrumentation have been rapidly declining when considered against the incentive to increase DER hosting capacity. Lastly, measurements and control signals from/to individual DERs to the intra-layer controller is the domain with the most diversity in communication requirements. Consider the range of operational metrology originating from devices as varied as residential smart meters to IEEE 2030 compliant PV inverters.

In our evaluations of the scalability and interoperability of DSO communications, we adopted an existing communication and information model for substation based SCADA systems: The International Electrotechnical Commission (IEC) 61850 series of protocols and model templates. This standard is frequently used for RTACs and similar devices, the standard also includes interfaces for phasor measurement units and streaming sampling sensors. Also, IEC 61850 supports the compositional communication model for intra-feeder and inter-feeder data exchange assumed in this work. Prior analyses on the protocol show that it meets the performance needs of SCADA based DSO control activities [62].

Returning to the representational needs for our work, an electrical (LV, MV or HV) substation with digital controls and communication capabilities is adequately expressed using the information models in the IEC 61850 group of standards [65]. The communication performance requirements for inter-substation relaying and SCADA telemetry are also well represented [66]. The generic object-oriented, building block for representing function elements, per the standard, is called a “logical node.” The logical nodes may be grouped in a multi-level hierarchy, which aligns with the proposed control hierarchy in this work. Recently, IEC 61850-7-420 (*Communications systems for DERs-Logical nodes* [67]) has been developed to extend the generic logical node model to DERs and the variety of communication services needed to support high-speed performance applications for both client-server and peer-to-peer DER communications. This harmonization of protocols and information models is relevant to our work since it enables the seamless representation of the *DER-to-VB-to-Substation-to-DSO* aggregation, while also appropriately describing the diverse communication needs at the DER level, the STL, and the FOL.

Thus, a SCADA power measurement at the head-node substation can be disaggregated and relayed to each service-level transformer in the STL, which in turn computes the tracking error. The embedded computer in the service transformer also runs the IEC 61850 logical node model for the VB interface at that location and hosts the communication interface to communicate with the DERs in the secondary (LV) network through protocols such as IEC 61850-7-420. The costs associated with the STL’s interface with DERs at the service-level transformer can either be rolled into VB technology service fees or DSO-deployed device charges and is not expected to represent a significant cost burden. The benefit of the embedded intelligence at each service transformer is a practically viable fast (100 to 500 ms loop time) intra-feeder control scheme and a variety of STL-DER interfaces that interoperate with existing substation SCADA protocols. Fast-acting intra-feeder control also ensures temporal decoupling from the GML and FOL that operate on a minutely timescale. Existing SCADA interfaces in a substation serve as the medium for all necessary data exchanges between STL and FOL (e.g., power flow optimization algorithms need updated load flow state information every 1 min to 5 min) to support corrective inter-feeder control. Next, we leverage these available communications to enable feedback control between layers.

### 7.2. Feedback Control between Layers

Due to the variable nature of PV generation, there are short-term fluctuations in net-demand constituting disturbances within a feeder. Hence, the slow time scale operation of the FOL optimization and subsequent dispatch of those set-points using the STL may not be sufficient to ensure that the head node power is tracked in real-time. In such a case, the flexibility available from the DERs can be used to mitigate these intra-feeder disturbances, by correcting the set-points provided by the FOL optimal VB set-point dispatcher. Furthermore, for a utility with multiple feeders connected to a substation, one feeder may suffer from larger disturbances, e.g., forecast errors not accounted for in the FOL OPF problem, cyberattacks on the DERs’ communication channels, and changes to network topology from local outages. In these cases, it becomes important for the system to be resilient [68] and maintain the economic set-point provided by the market despite such inter-feeder disturbances. In this section, we present a resilient and corrective control mechanism for mitigating these intra-feeder and inter-feeder disturbances by leveraging the flexibility of DERs. This ensures that feeders with high penetration of solar PV can be effectively dispatched to provide energy market services without sacrificing reliability.

#### 7.2.1. Inter-Feeder Control System

**Figure 10.**
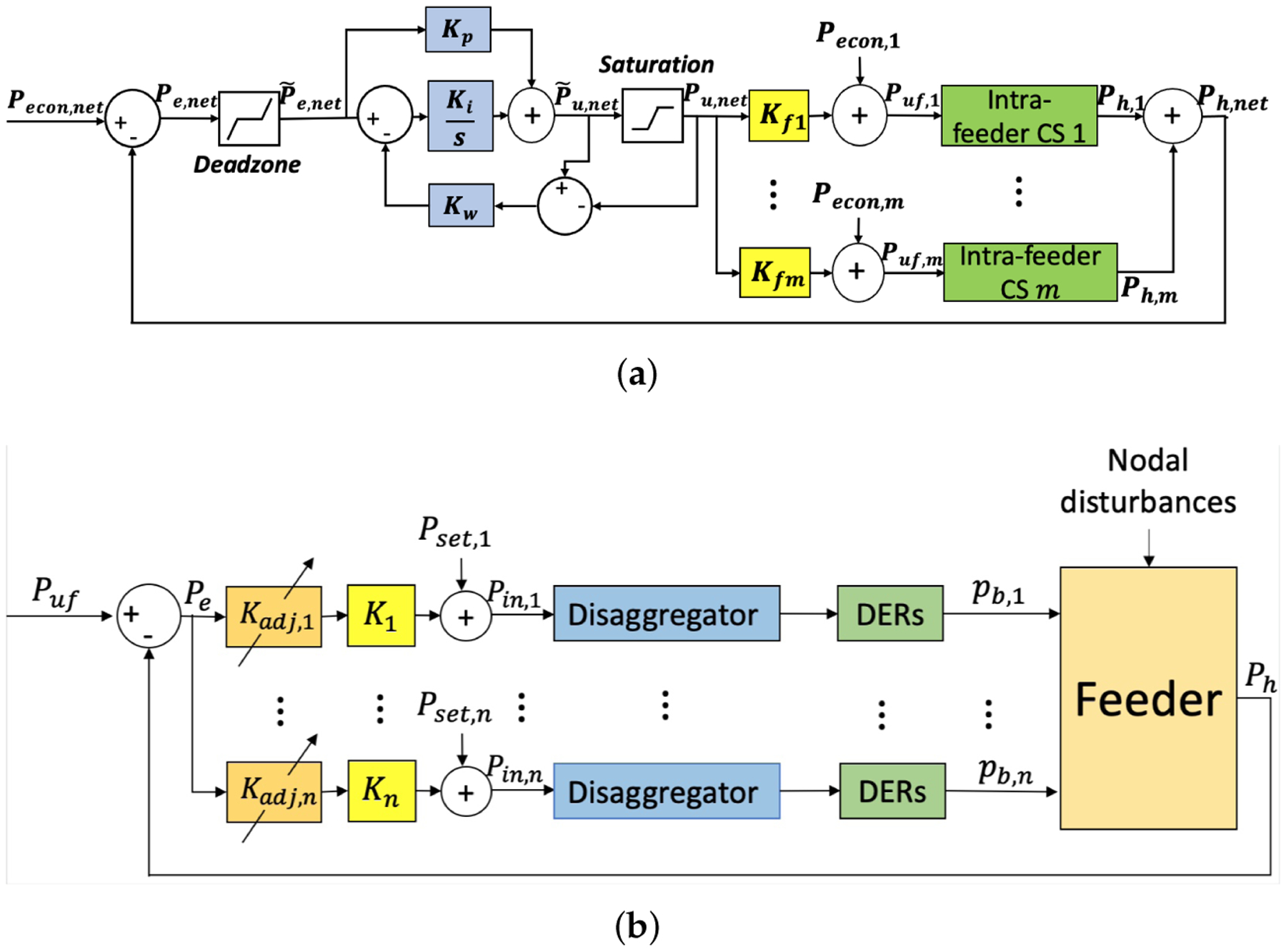
Hierarchical Real Time Control Scheme. (**a**) Inter-feeder Controller. (**b**) Intra-feeder Controller.

The inter-feeder control system mitigates large disturbances occurring within and across feeders and is depicted in Figure 10a. It is essentially a PI control scheme with a dead-zone and anti-windup mechanism that corrects the GML economic set-point references, *P*_econ,*r*_, to the *m* intra-feeder control systems (Figure 10b). The inter-feeder control system requires SCADA measurements of power flow from the head node of all connected feeders. These measurements are updated and control action taken every 5 s to accommodate communication and control response latency.

The working principle of the inter-feeder controller is as follows: The sum of measured head node active powers from all feeders, denoted by *P*_h,net_, is compared with the total economic market set-point for all feeders, *P*_econ,net_, and the error between them is passed through a dead-zone filter and PI controller with anti-windup. Then, the control input to the *r*th intra-feeder control system, *P*_*uf*,*r*_, is computed as *P*_*uf*,*r*_ = *K*_*fr*_*P*_*u*,net_ + *P*_econ,*r*_, where *P*_econ,*r*_ is the economic set-point for the *r*th feeder, *K*_*fr*_ is a scaling factor, and *P*_*u*,net_ is the output of the PI controller (with appropriate saturation limits).

The PI gains, *K*_*p*_ and *K*_*i*_, are selected considering requirements on settling time of response and stability, using a linearized model of the system, with the base demand, network parameters, and the FOL set-point as the “operating point”. The gains are updated about every 5 min to take into account changes in the system. The scaling factors *K*_*fr*_ can be chosen to penalize the extraction of power from feeders with lower capacity to supply power. Specifically, $K_{fi}={\bar{P}}_{i}/\bar{P}$, where ${\bar{P}}_{i}$ is the power capacity of the *i*th feeder, and $\bar{P}$ is the total power capacity of all feeders.

#### 7.2.2. Intra-Feeder Control System

The intra-feeder control scheme rejects short-term disturbances (like solar PV/demand fluctuations) that enter the primary MV nodes of a feeder and maintain the FOL set-point at the feeder’s head-node (i.e., substation). This controller is designed to have a loop time on the order of a few hundred milliseconds. To control the DERs in this scheme, the only measurement required is the active power at the head-node of the feeder, which is available at the substation, e.g., via RTACs. The scheme is depicted in Figure 10b, and, essentially, consists of a bank of proportional controllers, *K*_*r*_, multiplied by certain dynamic adjustment factors, *K*_adj,*r*_, one to control each of the *n* groups of DERs in the feeder. The corrected economic reference head-node power for this feeder, *P*_*uf*_, is calculated by the inter-feeder controller described in the previous subsection, and *P*_*h*_ the head node power of the feeder. Uncontrolled nodal disturbances are assumed to enter the feeder at multiple sites, unknown to the controller. The corrected set-point for the *r*th set of DERs, *p*_in,*r*_, is obtained as *p*_in,*r*_ = *K*_*r*_*K*_adj,*r*_(*P*_*uf*_ − *P*_*h*_) + *P*_set,*r*_, where *P*_set,*r*_ refers to the set-point provided by the FOL optimal set-point dispatcher about every minute, and *K*_adj,*r*_ is an adjustment factor that ensures that as the energy states of the VBs corresponding to the sets of DERs approach full capacity, the charging rate is proportionately reduced, and when the energy state becomes empty, the discharging rate is proportionately reduced (similar to standard gain scheduling). This helps to avoid a sudden step-change in power to zero when the DERs saturate (either empty or full capacity). The “disaggregators” then recast *P*_in,*i*_ as ON/OFF signals for individual devices using the optimization-based dispatch described in Section 6. Finally, the proportional controller gains, *K*_*r*_ can be designed using an optimal control approach, for example, one which minimizes the sum of squares of the tracking error and weighted control inputs to each set of DERs using the linearized model of the system (similar to what is considered for designing the inter-feeder control system gains). The gains are updated every 1 min to 5 min to consider changes in system parameters.

### 7.3. Proof of Concept: Inter-Layer Feedback Control

**Figure 11.**
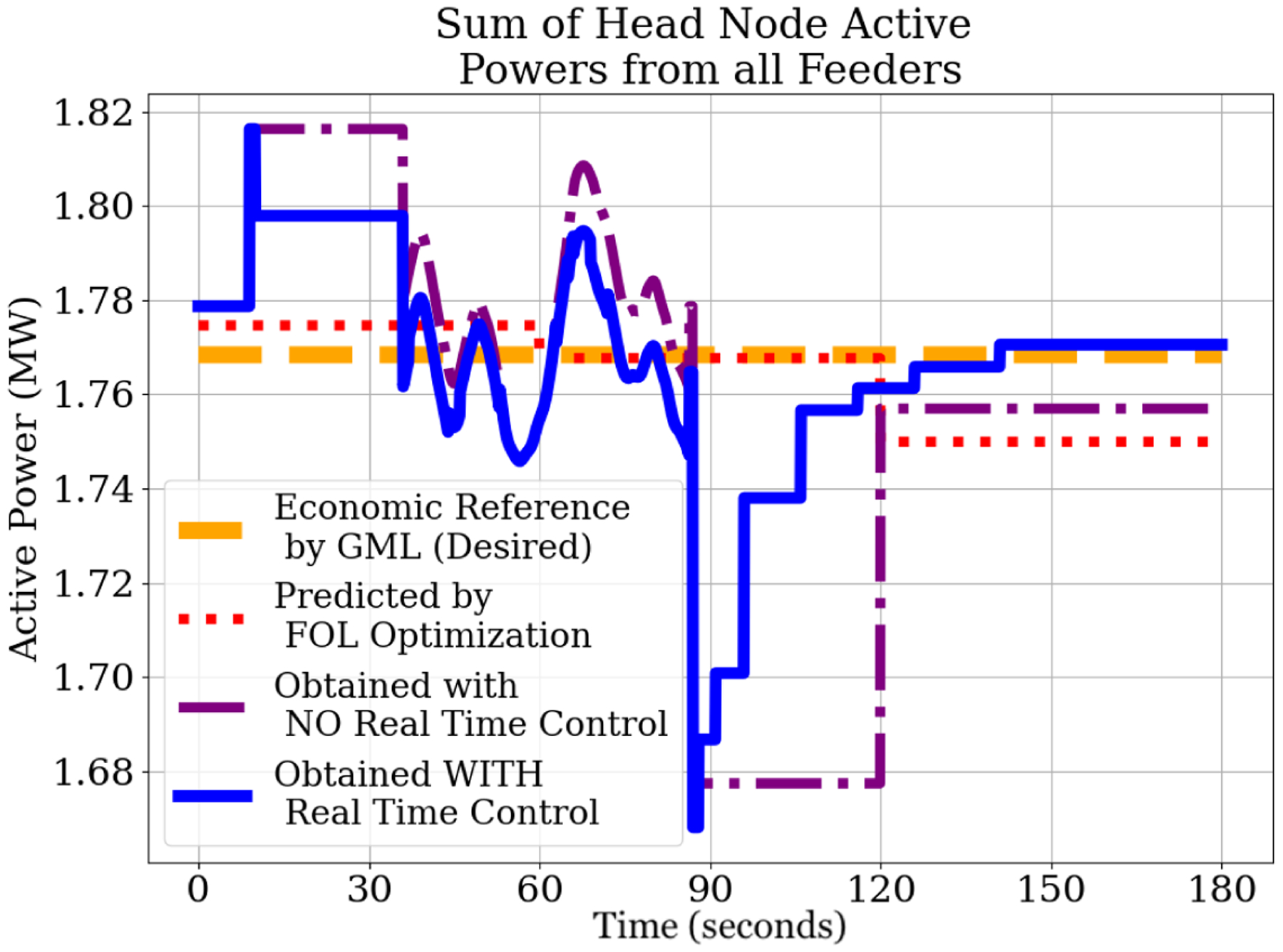
Simulation of intra- and inter-feeder controllers correcting static set-points to improve tracking.

In this subsection, we illustrate the performance of both the intra-feeder and inter-feeder control systems via simulation. Consider a simplified GML that provides economic set-points to two IEEE-37 node feeders (modeled using single-phase equivalents) and a simplified FOL with batteries representing VBs. Three sets of DERs, specifically containing 28, 28, and 26 water heaters, are assumed to be present in each feeder at different locations (specifically, where the base demand is 140 kW, 140 kW, and 126 kW respectively, to match the total rated power of the devices) making a total of 164 water heaters, each of rated power 4.5 kW. Under these simplified conditions, Figure 11 shows the results of a 3-min simulation to illustrate the effectiveness of inter-layer feedback control in rejecting disturbances while tracking the GML economic reference (shown as a yellow dashed line). We showcase three examples of disturbance classes that can be mitigated using the intra-feeder and inter-feeder controllers. First, at around 8 s, a step disturbance (e.g., due to persistent cloud cover) is added to some nodes of both the feeders. Since the change in total head node power is less than the dead-zone limit of the inter-feeder controller (assumed to be 72.7 kW, which is 10% of the total base demand in one feeder), only the intra-feeder controller remains active. The intra-feeder controller, combined with the optimization-based device-level dispatch, updates once every second and improves the tracking of the economic reference compared to the case where there is no real-time control (purple dash-dotted line). Second, at around 35 s, random noise is added (e.g., due to intermittent cloud cover). It can be seen that with real-time control, the variance of the total head node power is reduced. Finally, at around 88 s, 2 sets of DERs in the second feeder are assumed to be unexpectedly set to ‘zero’ power perhaps via malicious cyber intrusion. Since this is a major contingency, and the power change is more than 72.7 kW, the inter-feeder controller becomes active. Acting with a loop delay of 5 s, it brings the total head node power close to the desired value using remaining active devices from both feeders using the optimization-based dispatch. Thus, the real-time control mechanism is demonstrated as effective in mitigating various classes of disturbances. Moreover, the computation of the real-time control action, including the optimization-based device-level dispatch, took a maximum of 23 ms per control action in the simulation. Computational delays seem to be significantly shorter than the expected control action every 1 s to 5 s.

### 7.4. Proof of Concept: Communications between Layers

Validating the latency, throughput, and data scalability challenges associated with information exchange between FOL, STL, and individual DERs is fairly challenging and best approached via high fidelity simulation on a real-time digital simulator. Noting that these network performance metrics are sensitive to the choice of communication protocol, data exchange formats, and information models; a simulation of intra-feeder control was supplemented with a ‘real’ implementation of the full stack of IEC 61850 communication protocols.

This method of evaluation was used to assess the real-time feasibility of intra-feeder control communications noting the particularly demanding 1 s loop time of the controller. The simulation consisted of a single FOL element represented as a radial distribution feeder with 60 connected DER assets distributed across 15 secondary distribution circuits. Each secondary distribution circuit featured a service transformer augmented with the communication interfaces described in Section 7.1 and hosted between 3 and 5 DER assets. The modeled DER assets were a mix of PV generators and dispatchable loads in the form of electric water heaters and HVAC units. The entire 60 DER simulation was executed on an Opal-RT OP5600 real-time simulator with 1 millisecond simulation time steps. The temporal scaling between the different computation elements ranges from a 10 ms control loop time for individual DERs, 4 s loop times for the disaggregating DER controller, and a FOL update every one minute. All these computational updates are locked to the same hardware clock, ensuring they are coherent with each other.

To interface the simulated components with a real implementation of the communication stack, each DER asset and each service transformer was modeled as an IEC 61850-7-420 logical node [69], receiving individual power setpoints and configuration settings from the DER dispatch algorithm in the intra-feeder controller. These measurements and commands were modeled as IEC 61850-7-2 data objects, exchanged as IEC 61850-8-1 generic, object-oriented substation events (GOOSE) [65]. The full model, including dynamic representations of the DER assets and the IEC 61850 components, was constructed using the Simscape Electrical library and connected to software drivers for IEC 61850-8-1 communication using the methods outlined in [70].

**Figure 12.**
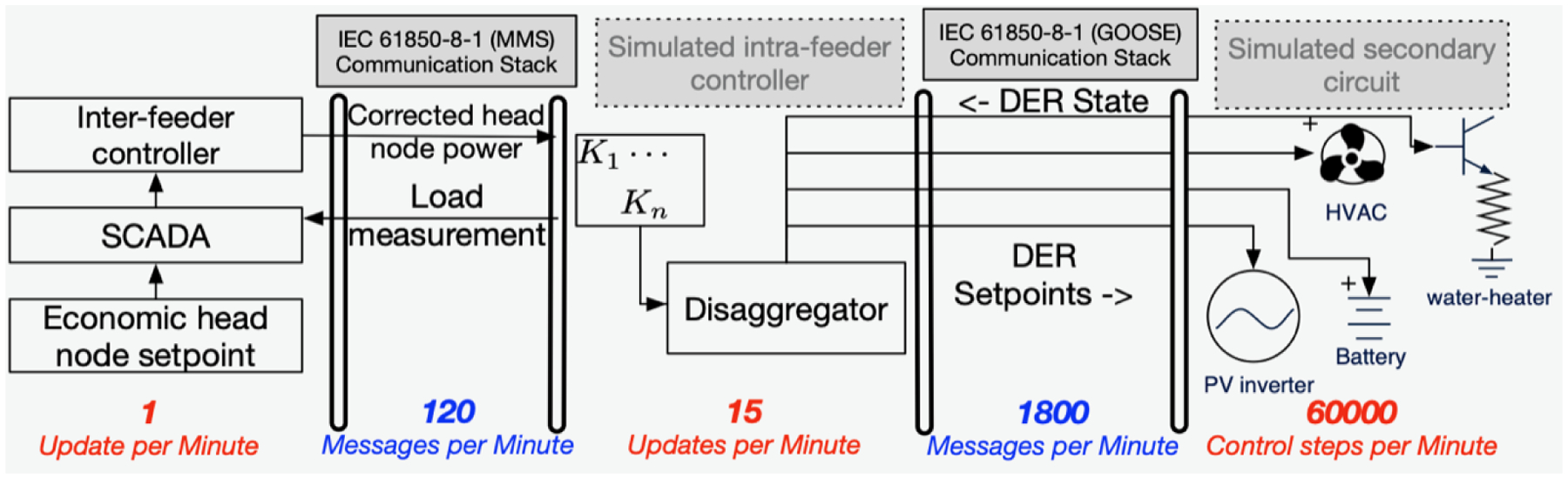
A schematic diagram showing the interacting elements in the validation environment. Here a single feeder and service transformer unit are considered. A total of 60 DER assets are coordinated by the system using International Electrotechnical Commission (IEC) 61850 compliant information models and communication protocols.

An overview of the validation setup is shown in Figure 12. 45 DERs receive ON/OFF commands as IEC 61850-8-1 compliant *DRCC*/*DERStr* and *DRCC*/*DERStop* events. The 15 remaining DERs (assumed to be dispatchable PV generators) are provided power curtailment requests as *DRCC*/*WSet* events. 1800 IEC 61850-8-1 GOOSE messages per minute are generated to meet the DER control loop time. The intra-feeder controller in turn interacts with the inter-feeder controller and the FOL using IEC 61850-8-1 Manufacturing Message Specification (MMS) messages, there are 120 corrected head node power setpoints received per minute.

The validation aspect of this exercise was establishing whether potential congestion from rapid, periodic updates would adversely affect the performance of the control system at the STL. All IEC 61850 messages produced by the simulation were routed through a data pipeline complete with network routers and data processing buffers designed so that the pipeline would introduce realistic communication delays and errors.

**Figure 13.**
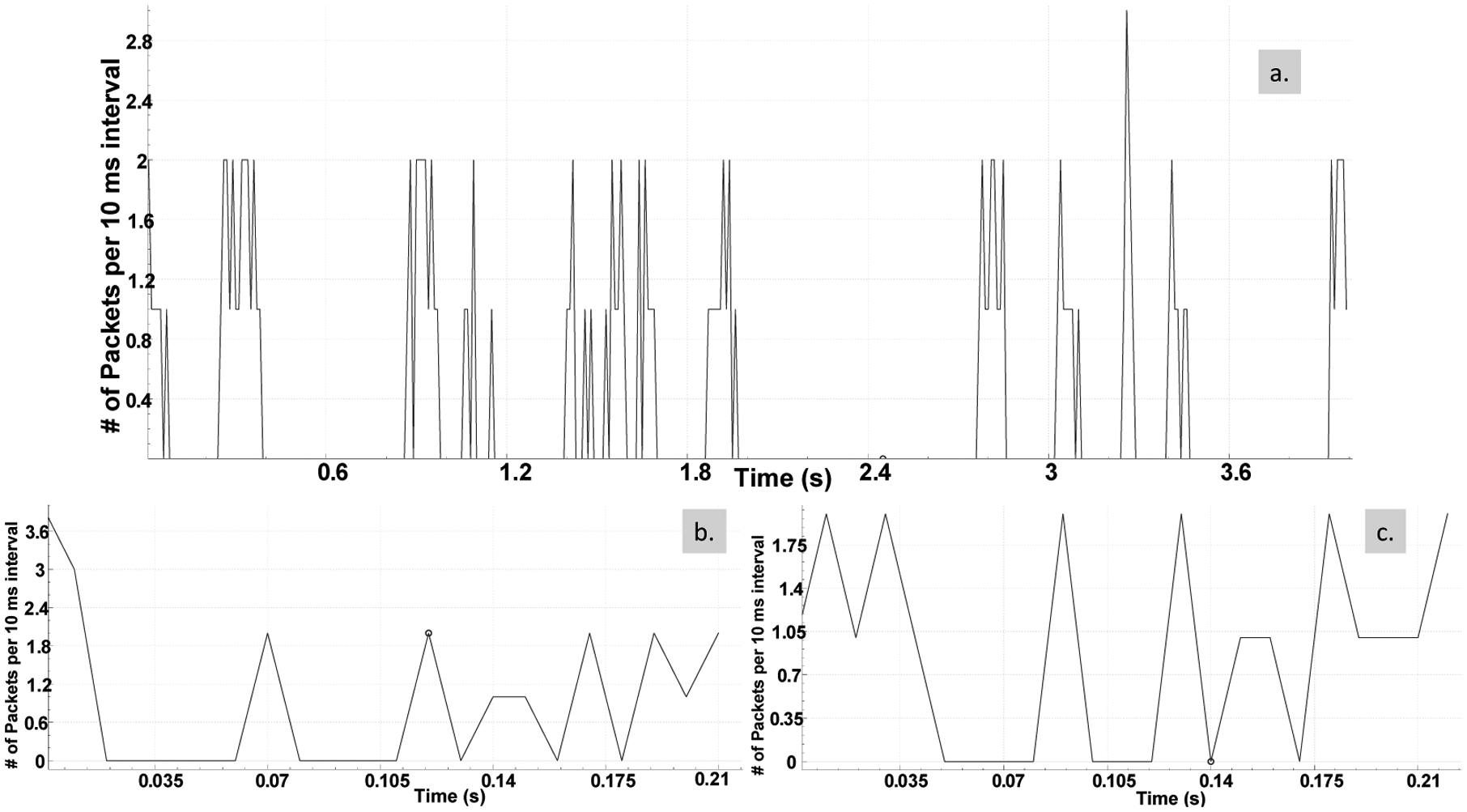
Communication throughput: (**a**). Generic, object-oriented substation events (GOOSE) exchanges between STL and DERs (average latency ≈5 ms). (**b**). Load request correction from FOL to STL (average latency ≈ 10 ms). (**c**). Load estimate query from STL to FOL.

Figure 13 shows the throughput of the data pipeline while performing this experiment. Figure 13a shows a histogram of packet arrival rate. The x-axis on the plot shows the total time taken for all 60 DERs to transmit their state and receive commands from the STL. In this particular instance, all 60 DERs were updated in under the update loop time for the STL of 4 s. The average latency of the updates was 5.2 ms which is well below the requirement of 100 ms discussed earlier. Similarly, Figure 13b,c show the stable throughput exceeding the update constraints of the FOL with bounded message latency of approximately 10 ms.

Thus, the proof of concept validation presented in this section suggest that the necessary inter-layer communications and closed-loop inter- and intra- feeder control are practically implementable in real-time and provide robustness against exogenous disturbances. The next section investigates how the three layers (GML, FOL, and STL) work together to achieve a system-wide task (peak demand reduction), while managing high solar PV penetration along with grid and device constraints.

## 8. Large-Scale Coupled Simulation Results

While the previous section highlighted the practical viability of real-time feedback control and communications between the different layers, this section focuses on large-scale simulation and validation of the coupled GML-FOL-STL-DER energy resource hierarchy. Specifically, we consider a GML that dispatches more than 100 FOL elements across its NYISO sub-transmission network and three of these FOL elements embody fully modeled, industry-provided, unbalanced feeders. Each fully-modelled feeder is Kron-reduced and have 100 to 200 STL elements at super-nodes with corresponding VB parameters and for which 1 to 2 STL elements in each feeder are fully populated with groups of DERs and solar PV inverters. To illustrate coordination across the three-layer hierarchy on this large system, we propose to fully model out the system under GML’s Scenario #3 from Section 4.5, which considers the valuable market service: peak demand reduction (i.e., peak shaving), which costs the DSO $10,000/MW-month for the peak day. As in Scenario #3, the GML will also participate in the (less valuable) real-time market during this day to illustrate multi-timescale grid services. This economic scenario is interesting because it creates system-wide economic benefits but requires careful coordination to reduce the system peak demand over a day. In addition, the DSO’s system includes high levels of solar PV penetration (i.e., 50% of energy demand is supplied from solar PV), which represents a challenge to reliable grid operations of each feeder. GridLab-D is utilized to conduct the full-scale feeder and DER simulations.

### 8.1. Simulation Setup

**Figure 14.**
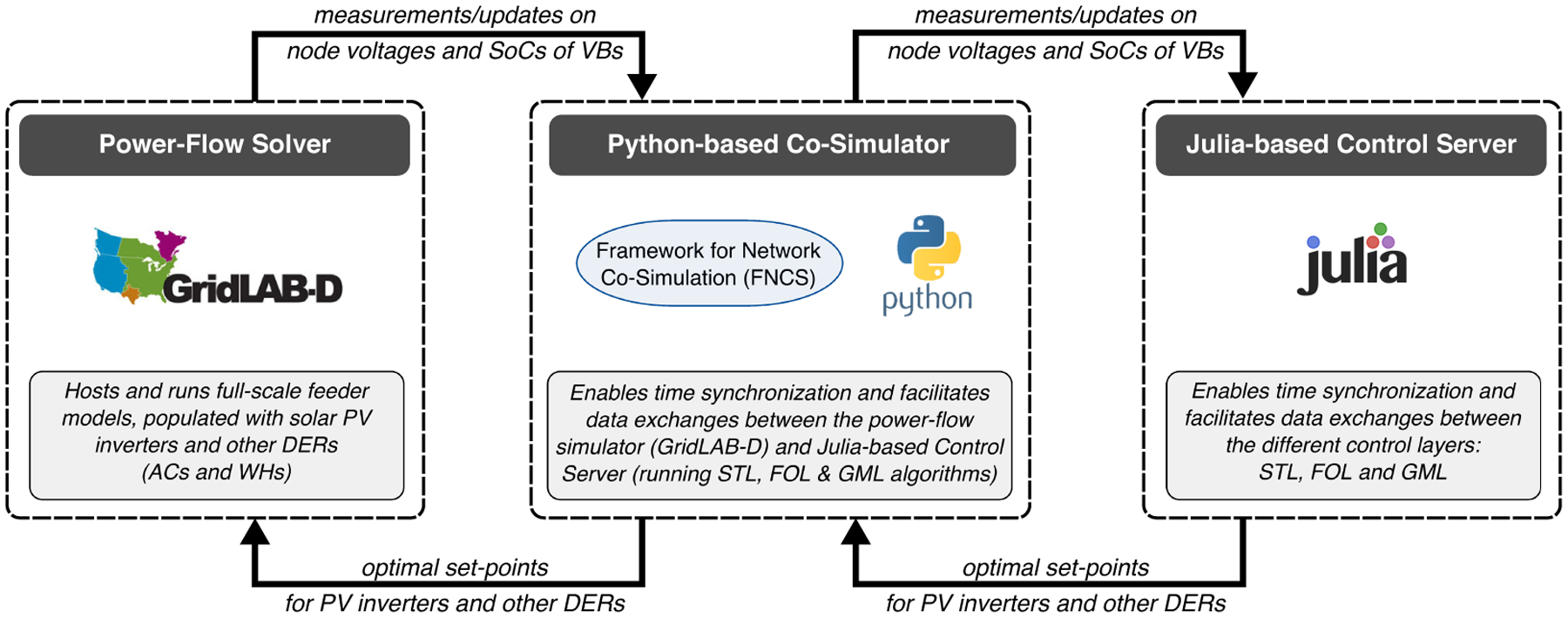
The integrated co-simulation environment for numerical validation of the coupled hierarchical stochastic control algorithms (STL, FOL and GML) with large-scale simulations of distribution feeder models populated with solar PV and other DERs.

This section provides a brief overview of the simulation environment used to test the algorithms. Figure 14 shows the overall framework used in the simulations. The simulation demonstrates the efficacy of the proposed framework on a 79-bus meshed sub-network of NYISO’s sub-transmission system, where each load bus represent a transformer bank with 4 to 8 feeders for a total of more than 150 feeders. Three feeders at three different buses in the network are fully modelled three-phase unbalanced circuits in Gridlab-D. The three feeders represent primary radial distribution networks with 1213, 936 and 594 nodes, respectively, and are Kron-reduced into 125, 90 and 60 super nodes, respectively. Each super-node represents an STL element with corresponding VB parameters. The virtual battery models developed for DERs (as detailed in Section 6) are incorporated into the GridLab-D model. Given the VB parameters, a subset of the STL nodes (representing a super node in the reduced model) in each of the feeder are populated with air-conditioners, electric water heater, and solar PV inverters representing the available distributed flexibility and generation. The other super-nodes are augumented with a Gridlab-D battery object with capacity and power rating identical to the corresponding virtual battery parameters. The PV inverters were slightly oversized to provide some reactive power flexibility. The full feeder along with the newly created batteries and inverters at the corresponding super nodes in the full network formed the final GridLab-D model used in the simulations.

The framework requires integration and complex interactions between the FOL, GML and STL algorithms and the GridLab-D model. To enable time synchronous simulations of the different layers, we employed the Framework for Network Co-Simulations (FNCS), which is an open-source co-/multi-simulation framework, which uses a federated approach for integrating multiple simulators.

Exchange of information, including synchronization of simulator clocks among grid and market simulators will be maintained using central agent called the FNCS broker. Since FNCS has the capability of co-/multi-simulations of multi-domain simulators, it provides an efficient solution to synchronize the different algorithms with the Gridlab-D model. Also, FNCS can synchronize multiple packages, tools, and simulators hosted in different machines, which ultimately ensure modular and parallel development and integration of different packages.

FNCS receives information from the GridLab-D model such as the battery state of charge needed for the FOL algorithm. The information is exchanged in the form of key-value pairs which then have to be parsed before being communicated to the algorithms. This is done using a Python API which parses the data received from FNCS before sending them to a Julia server that contains the GML, FOL and STL algorithms. Once the Julia server receives the data from Python, DSSE uses the voltage measurements to compute the state of the system. The FOL algorithm uses the battery state of charge values and the GML tracking signal to compute the new active and reactive power set-points of the inverters. These values are returned back to the Python API which then converts it into key-value pairs before passing it back to FNCS. FNCS then relays this information to GridLab-D which updates the inverter set-points, thereby completing the loop.

### 8.2. Results

**Figure 15.**
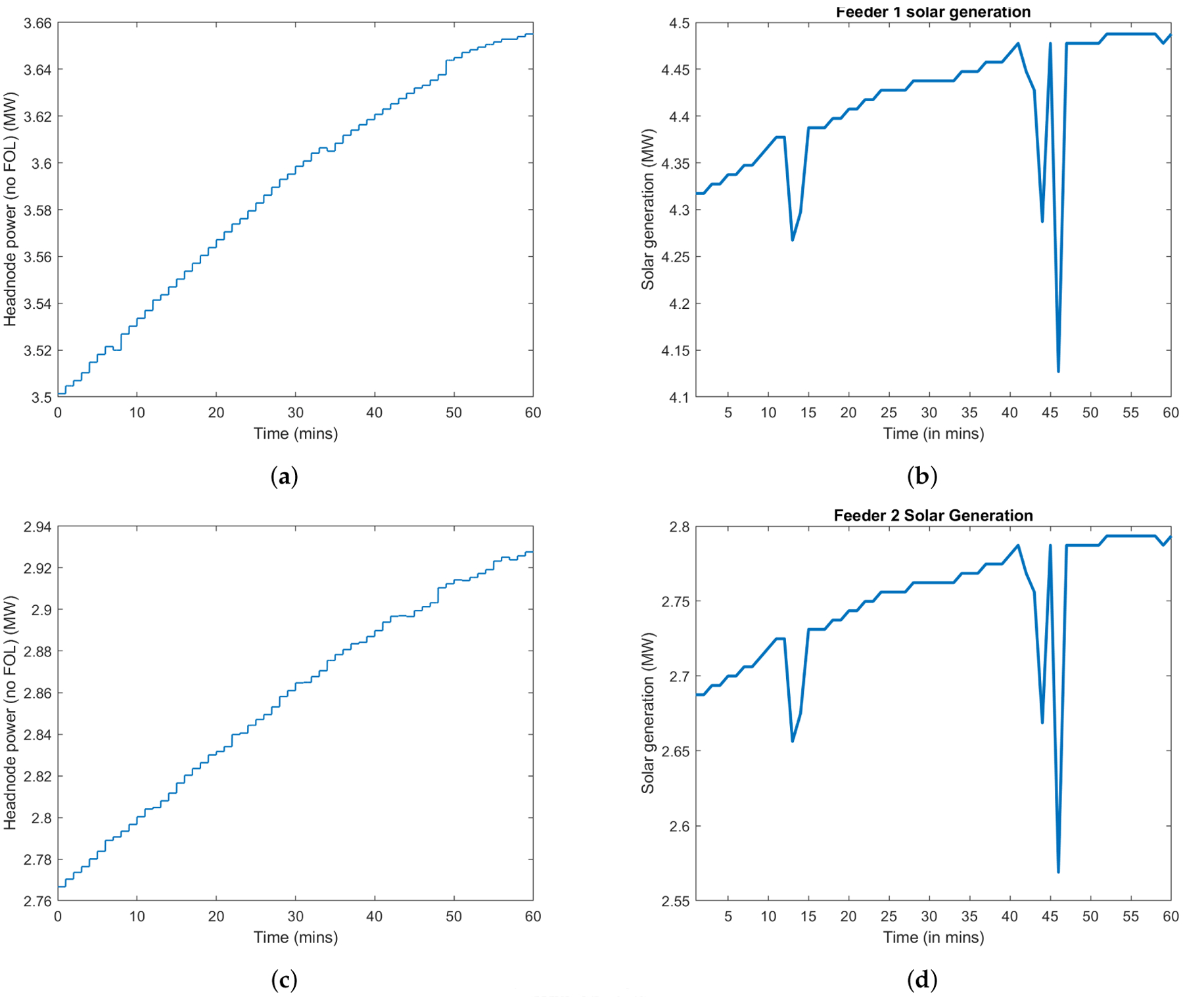

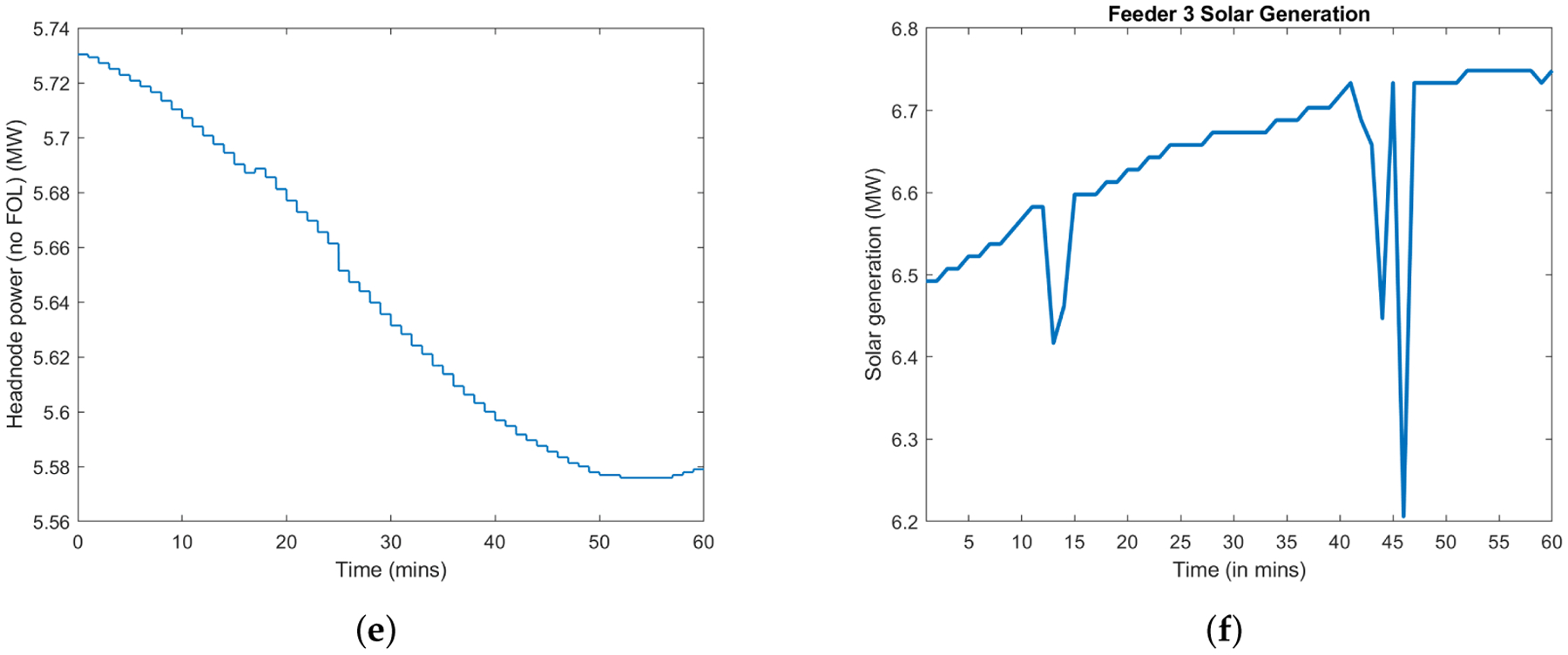
Total nominal active power demand and supply at the headnode of each feeder. (**a**) Feeder 1 nominal head node demand. (**b**) Feeder 1 available solar PV generation. (**c**) Feeder 2 nominal head node demand. (**d**) Feeder 2 available solar PV generation. (**e**) Feeder 3 nominal head node demand. (**f**) Feeder 3 available solar PV generation.

The simulation was conducted for one of the system’s annual peak demand hours (1 August, 11:00 a.m.–12:00 a.m.). The nominal headnode active power demand and total available solar PV supply profiles for the fully modelled feeders is shown in Figure 15. The simulation scenario considered herein is that of peak shaving, where the maximum demand is reduced for a day.

#### 8.2.1. Peak Shaving

**Figure 16.**
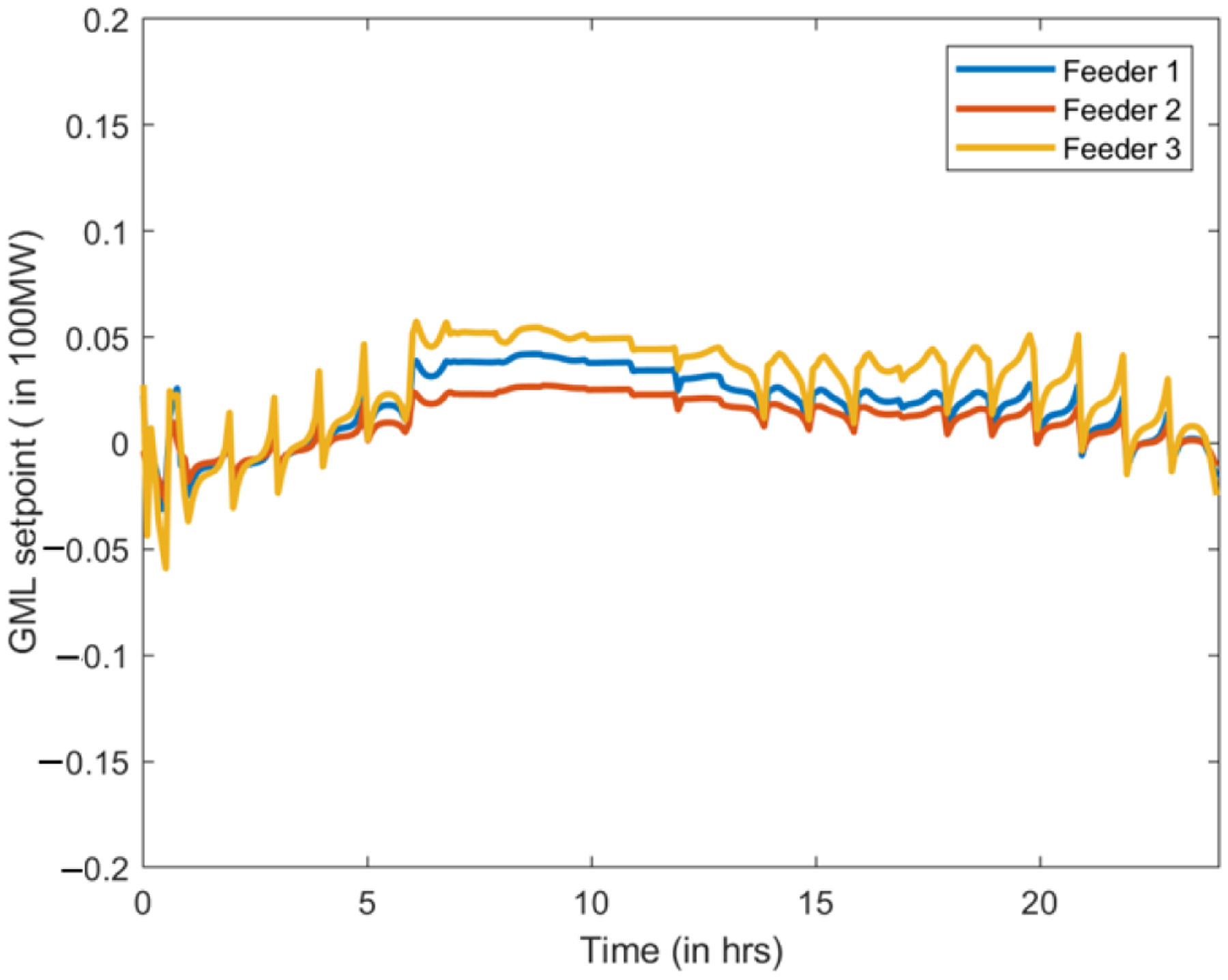
The 24-h GML real power set-point trajectory for three fully modelled feeders starting from 11:00 in the peak-shaving mode.

The GML operates in *real-time with peak shaving mode* detailed in Sections 4.4 and 4.5 as *Scenario #3*. The VBs are initialized at 50% state of charge (SoC). The aggregate VB capacity in each of the feeders is listed in Table 3. The GML setpoint for the next 24 h (starting from 11:00 a.m.) for all of the fully modelled feeders is shown in Figure 16.

**Figure 17.**
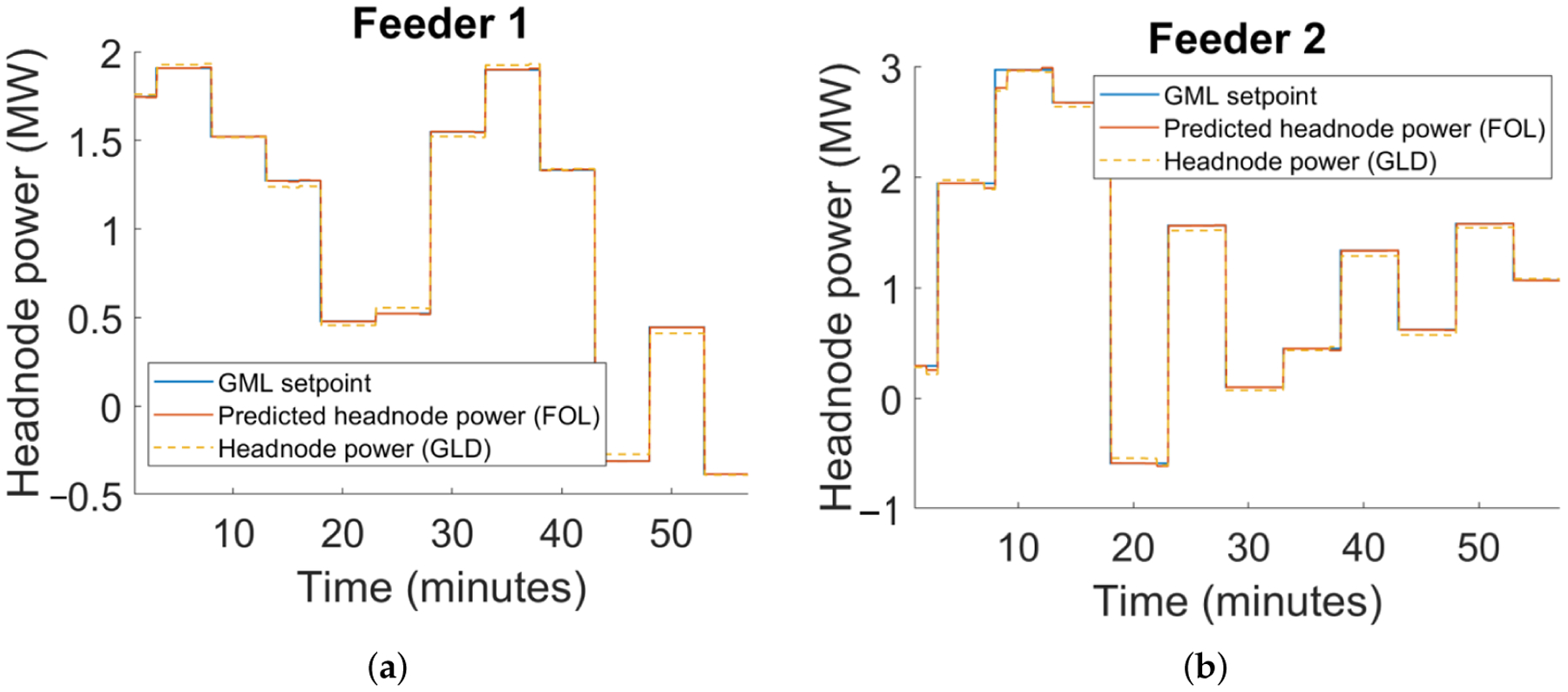

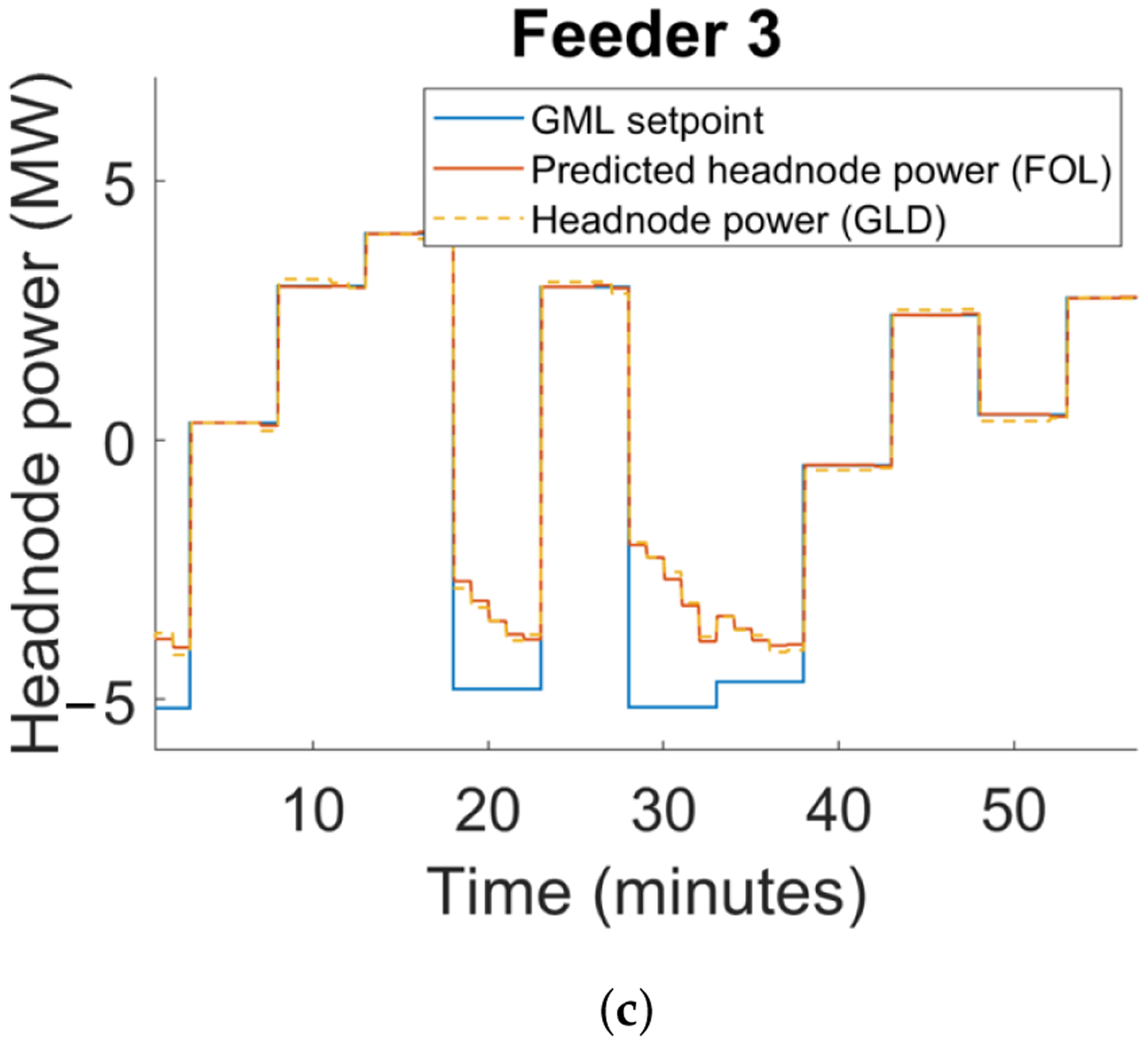
GML setpoint tracking performance for the fully modelled feeders in the FOL. (**a**) Feeder 1. (**b**) Feeder 2. (**c**) Feeder 3.

**Figure 18.**
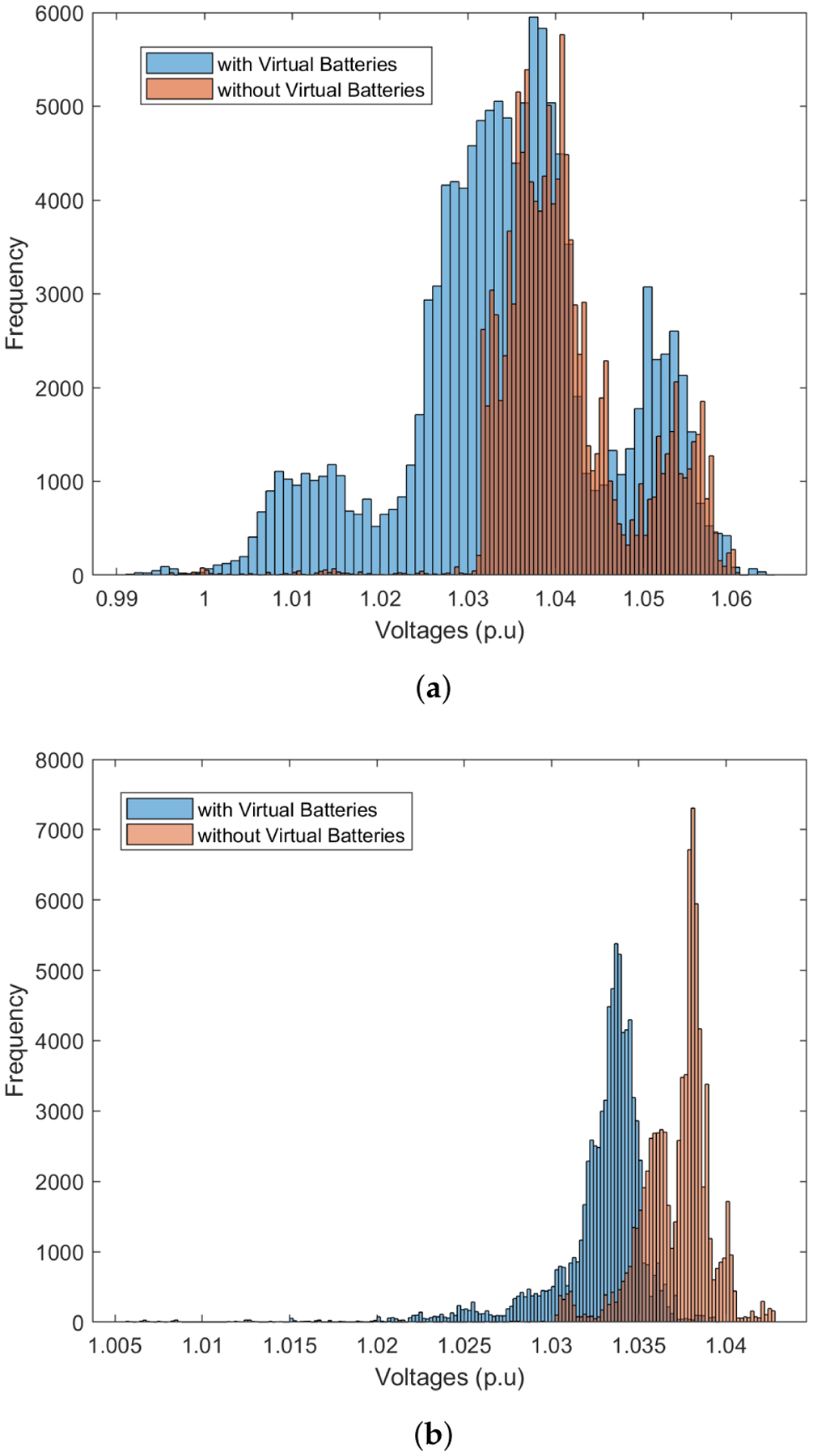

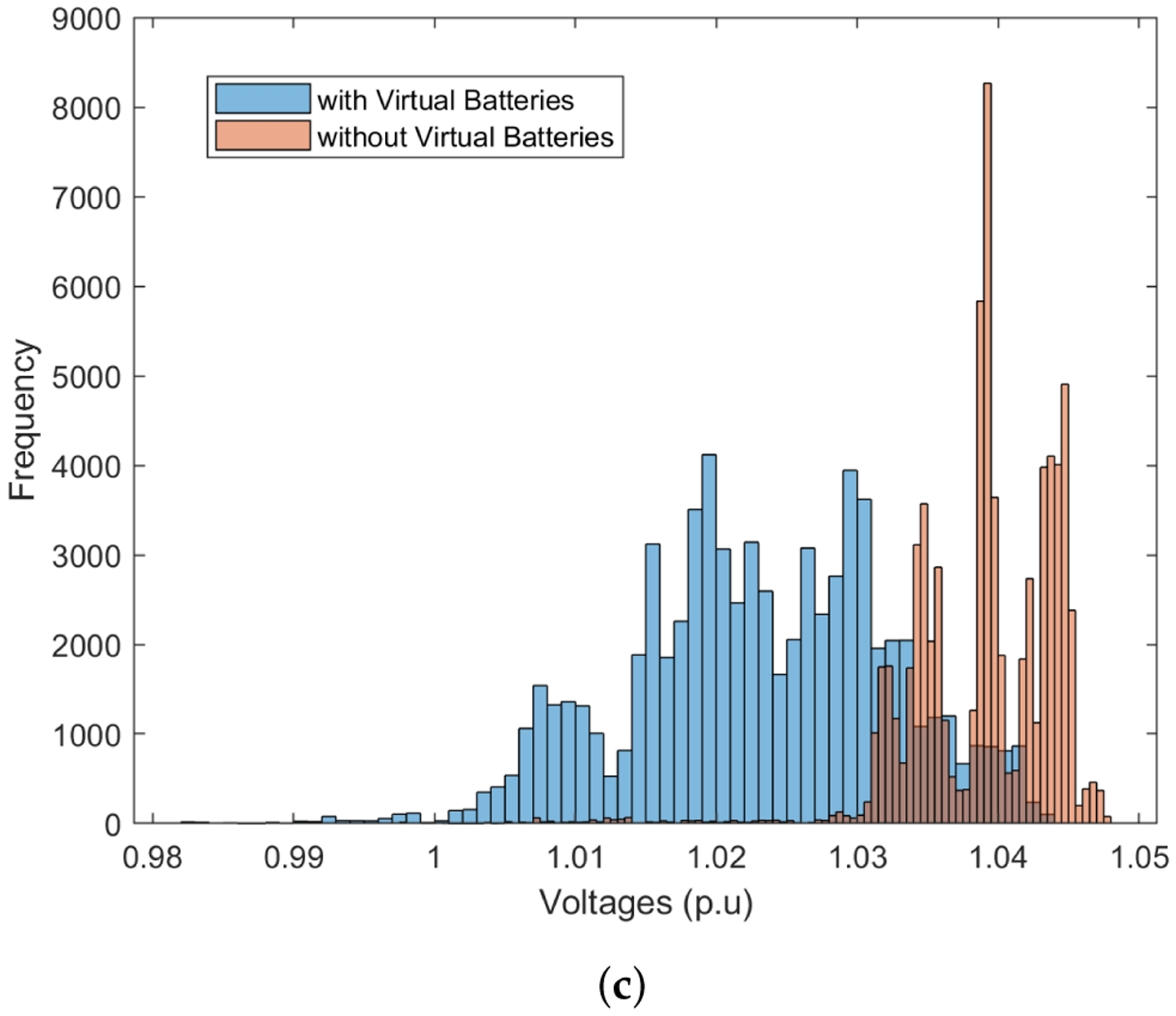
Distribution of voltage magnitudes for all nodes during peak hour (11:00 to 12:00). (**a**) Feeder 1. (**b**) Feeder 2. (**c**) Feeder 3.

**Figure 19.**
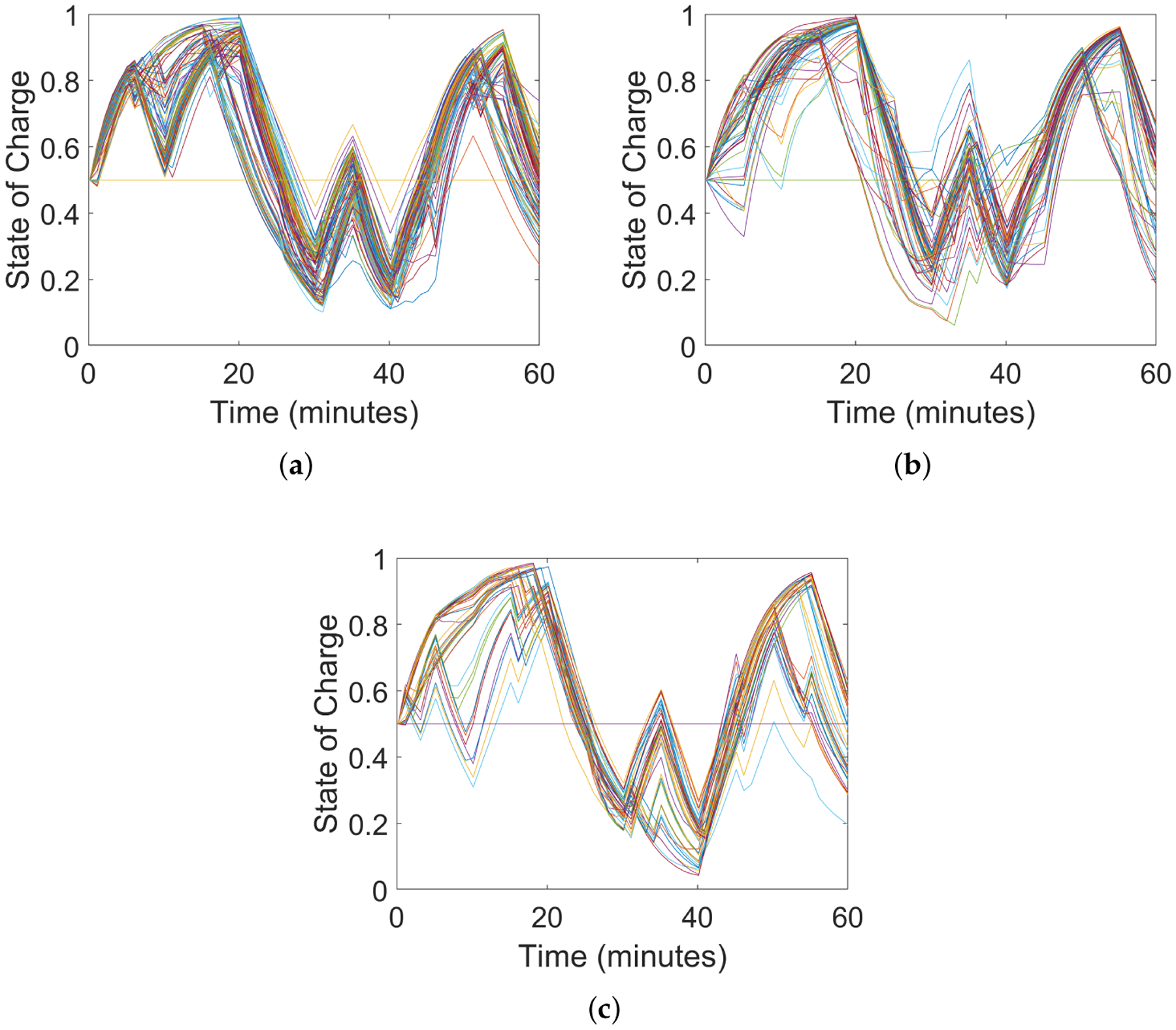
Evolution of the VBs’ normalized state of charge during peak hour in the FOL. (**a**) Feeder 1. (**b**) Feeder 2. (**c**) Feeder 3.

**Figure 20.**
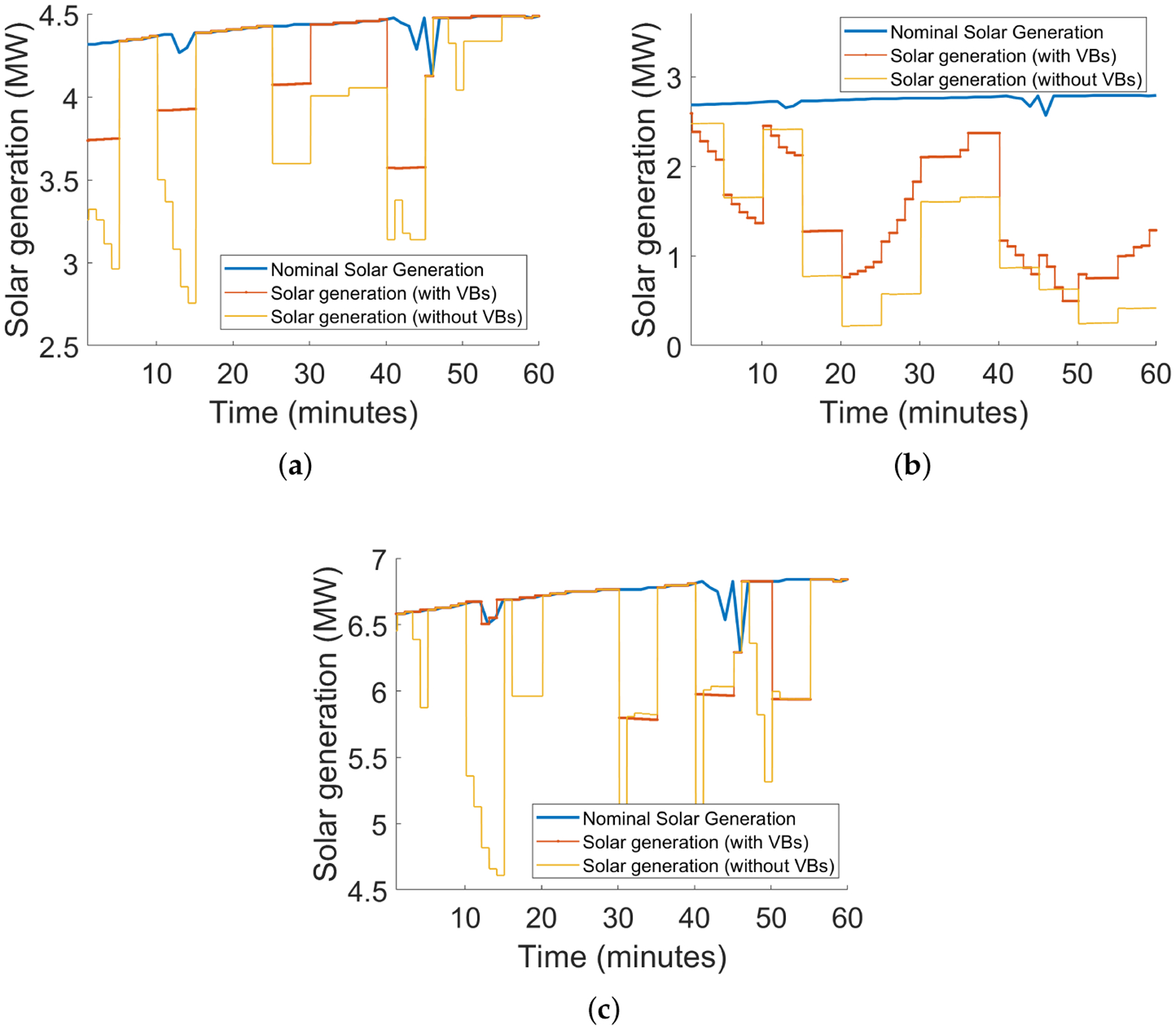
Total solar PV output from all nodes during peak hour (11:00 to 12:00) in the FOL. (**a**) Feeder 1. (**b**) Feeder 2. (**c**) Feeder 3.

Figure 17 shows the FOL tracking performance across all the three feeders and is quantified in Table 4. Overall, the tracking errors for each fully modeled feeder ranges from 14–90 kW (RMSE), which is low relative to the MW-scale net-load of the feeders. However, feeder 3’s larger tracking error in Figure 17c is due to the GML forecasting far more solar PV from feeder 3 than is realized at time 20 and 35 min, which drives up the net-demand at the head-node. Had the inter-feeder controller been active in this simulation, we would expect to see the other feeders correct their head-node set-points down to make up for the GML’s desired aggregate head-node demand. Nonetheless, the FOL is able to maintain the nodal voltage magnitudes within the ANSI bands with a probability of 0.95 with the use of VBs (Table 4). Figure 18 provides a comparison of the nodal voltage distribution with and without virtual batteries. The voltages tend to higher values in the absence of virtual batteries due to high solar PV penetration levels in all feeders. Figure 19 shows how VBs’ state trajectories evolve over the peak hour for all three feeders and represent the device-level energy/comfort constraints (i.e., no violation of [0,1] bounds implies no violation of device constraints). Given the full range of utilization of the VBs, it is clear that the FOL utilizes the available flexibility. The overall PV output during the peak hour is shown Figure 20 and illustrates how VB flexibility allows the FOL to reduce the level of solar PV curtailment and more closely track GML set-points (see Table 5).

Thus, from the large, coupled simulation study, we have shown that the novel hierarchical scheme enables large-scale coordination of DERs that ensure (1) system-wide economic objectives are met (i.e., GML set-points tracked; costs are minimized) while (2) reliably managing high levels of solar PV output and flexibility within each unbalanced feeder (i.e., voltages are well-behaved, reduced PV curtailment); and (3) satisfying device-level constraints on comfort/energy (i.e., VBs within limits).

## 9. Conclusions

A novel three-layer hierarchical approach to coordinating DERs has been presented that spatially and temporally decomposes electric distribution system operations to enable deep penetration levels of distributed solar PV generation and valuable grid services. This is achieved by coordinating DERs with the GML’s system-wide TSO/DSO market optimization interface while the FOL then actively manages reliability in unbalanced distribution feeders while tracking market signals. Finally, DERs are dispatched locally to realize the system-wide economic set-point with the STL, which also manages device-level energy constraints/requirements. Together, the three layers reshape net-demand based on market and grid conditions. The proposed approach leverages a utility-centric implementation that permits practically viable inter-layer communication and real-time responsiveness and control. In addition, a large-scale coupled simulation study involving all three layers suggests that a utility would benefit from the proposed hierarchy with improved reliability and economics, reduced curtailment of solar PV, and effective utilization of demand-side flexibility.

Future work will focus on studying performance guarantees within and between the different layers, which has not been developed. Specifically, the GML would benefit from optimality guarantees for convex OPF-based methods that have been employed to optimize the meshed sub-transmission networks. In addition, the proposed utility-centric approach places a large technological (data processing, communication, control) burden on the utility and limits participation of independent DER owners and aggregators, which creates regulatory challenges. Furthermore, given the optimization-based methods employed in the FOL and GML, the approach is also limited to slower (minutely) market signals and not suitable for dispatching controllable resources against fast TSO signals like frequency regulation (e.g., updated every 2–4 s by the TSO). To overcome these challenge, ongoing work is considering new optimization methods that implicitly embed AC grid constraints into nodal capacity bounds for controllable grid resources that then enable a real-time controller to determine a DER’s participation factor to a feeder’s desired power set-point [71]. This would also allow utilities to offer their (dynamic) hosting capacity as a market product to independent DER owners and aggregators and create new market-based services for flexible demand. The authors are also interested in extending the STL’s deterministic characterization of VBs to incorporate stochastic elements and more diverse DER populations, along the line of [26]. Finally, the simulation setup may be useful for exploring more scenarios that may be of value to utilities and regulators.

## Figures and Tables

**Table 1.** Economic benefits of grid market layer (GML).

	Without VB	VB: 75 MW + 187.5 MWh		VB: 150MW + 375 MWh	
Scenario	#*1*	#*2*	#*3*	#*2*	#*3*
Real-time cost ($)	428,330	425,981	426,053	424,322	424,486
Solar curtailment cost ($)	0	0	0	0	0
Peak cost ($)	12,609,000	13,299,920	12,150,240	14,061,360	11,881,330
Total cost ($)	13,037,330	13,725,901	12,576,293	14,485,682	12,305,816

**Table 2.** Per-unit savings for VB capacity and power rating.

	VB: 75 MW + 187.5 MWh		VB: 150 MW + 375 MWh	
Scenario	#*2*	#*3*	#*2*	#*3*
Real-time saving	12.59 $/MWh	\	10.72 $/MWh	\
Peak saving	\	2448.80 $/MWh	\	1941.49 $/MWh

**Table 3.** Aggregate virtual battery parameters for each feeder.

	Capacity (MWh)	Power Rating (MW)
Feeder 1	0.45	2.26
Feeder 2	0.29	1.45
Feeder 3	0.80	3.66

**Table 4.** Tracking root-mean-square error (RMSE) error and 95th percentile of the nodal voltage distribution when virtual batteries are utilized.

	Tracking RMSE	Voltage u.b. with VB (95th Percentile)	Voltage u.b. without VB (95th Percentile)
Feeder 1	14 kW	1.053 p.u	1.058 p.u
Feeder 2	20 kW	1.035 p.u	1.041 p.u
Feeder 3	90 kW	1.038 p.u	1.047 p.u

**Table 5.** Average curtailment with and without virtual batteries.

	Mean Curtailment with VB	Mean Curtailment without VB
Feeder 1	0.2 MW	0.5 MW
Feeder 2	1.2 MW	1.6 MW
Feeder 3	0.3 MW	0.6 MW

## References

[1] Lazard. Levelized Cost of Energy Analysis-Version 13.0; Technical Report; Lazard: Hamilton, Bermuda, 2019.

[2] United Nations Environment Programme. Emissions Gap Report; UNEP: Nairobi, Kenya, 2019.

[3] Goldenberg C; Dyson M; Masters H Demand Flexibility: The Key To Enabling A Low-Cost, Low-Carbon Grid; Technical Report; Rocky Mountain Institute: Bassault, CO, USA, 2018.

[4] Hledik R; Faruqui A; Lee T; Higham J The National Potential for Load Flexibility: Value and Market Potential Through 2030; Technical Report; The Brattle Group: Boston, MA, USA, 2019.

[5] IEA. Digitalisation and Energy; Technical Report; International Energy Agency: Paris, France, 2017.

[6] DeMartini P Future of U.S. Electric Distribution: Part II; Technical Report; Edison Electric Institute: Washington, DC, USA, 2012.

[7] Wu FF; Varaiya PP; Hui RS Smart Grids with Intelligent Periphery: An Architecture for the Energy Internet. Engineering 2015, 1, 436–446.

[8] Taft JD Architectural Basis for Highly Distributed Transactive Power Grids: Frameworks, Networks, and Grid Codes; Technical Report; Pacific Northwest National Lab. (PNNL): Richland, WA, USA, 2016.

[9] Taft JD Grid Architecture 2; Technical Report; Pacific Northwest National Lab. (PNNL): Richland, WA, USA, 2016.

[10] De Martini P Operational coordination architecture: New models and approaches. IEEE Power Energy Mag. 2019, 17, 29–39.

[11] Martini PD; Kristov L; Schwartz L Distribution Systems in A High Distributed Energy Resources Future: Planning, Market Design, Operation and Oversight; Technical Report; Lawrence Berkeley National Laboratory: Berkeley, CA, USA, 2015.

[12] Kristov L; De Martini P; Taft JD A tale of two visions: Designing a decentralized transactive electric system. IEEE Power Energy Mag. 2016, 14, 63–69.

[13] Kok K; Widergren S A Society of Devices: Integrating Intelligent Distributed Resources with Transactive Energy. IEEE Power Energy Mag. 2016, 14, 34–45.

[14] Nazir MS; Hiskens IA A dynamical systems approach to modeling and analysis of transactive energy coordination. IEEE Trans. Power Syst 2018, 34, 4060–4070.

[15] Consolidated Edison Company of New York. Consolidated Edison Distributed System Implementation Plan; Con Edison DSIP Filing: New York, NY, USA 2018; pp. 1–7.

[16] Molzahn D; Roald LA Grid-Aware versus Grid-Agnostic Distribution System Control: A Method for Certifying Engineering Constraint Satisfaction. In Proceedings of the Hawaii International Conference on System Sciences, Honolulu, HI, USA, 8–11 January 2019.

[17] Dall’Anese E; Guggilam SS; Simonetto A; Chen YC; Dhople SV Optimal regulation of virtual power plants. IEEE Trans. Power Syst 2018, 33, 1868–1881.

[18] Arnold DB; Sankur MD; Negrete-Pincetic M; Callaway DS Model-Free Optimal Coordination of Distributed Energy Resources for Provisioning Transmission-Level Services. IEEE Trans. Power Syst 2018, 33, 817–828.

[19] Bidram A; Davoudi A Hierarchical structure of microgrids control system. IEEE Trans. Smart Grid 2012, 3, 1963–1976.

[20] Baker K; Bernstein A; Dall’Anese E; Zhao C Network-Cognizant Voltage Droop Control for Distribution Grids. IEEE Trans. Power Syst 2018, 33, 2098–2108.

[21] Dörfler F; Simpson-Porco JW; Bullo F Breaking the Hierarchy: Distributed Control and Economic Optimality in Microgrids. IEEE Trans. Control Netw. Syst 2016, 3, 241–253.

[22] Hao H; Sanandaji BM; Poolla K; Vincent TL Aggregate flexibility of thermostatically controlled loads. IEEE Trans. Power Syst 2014, 30, 189–198.

[23] Hughes JT; Domínguez-García AD; Poolla K Identification of Virtual Battery Models for Flexible Loads. IEEE Trans. Power Syst 2016, 31, 4660–4669.

[24] Chakraborty I; Nandanoori SP; Kundu S Virtual battery parameter identification using transfer learning based stacked autoencoder. In Proceedings of the 2018 17th IEEE International Conference on Machine Learning and Applications (ICMLA), Orlando, FL, USA, 17–20 December 2018; pp. 1269–1274.

[25] Nandanoori SP; Chakraborty I; Ramachandran T; Kundu S Identification and validation of virtual battery model for heterogeneous devices. In Proceedings of the 2019 IEEE Power & Energy Society General Meeting (PESGM), Atlanta, GA, USA, 4–8 August 2019; pp. 1–5.

[26] Chakraborty I; Nandanoori SP; Kundu S; Kalsi K Stochastic Virtual Battery Modeling of Uncertain Electrical Loads Using Variational Autoencoder. In Proceedings of the 2020 American Control Conference (ACC), 1–3 July 2020; pp. 1305–1310.

[27] Nazir N; Racherla P; Almassalkhi M Optimal Multi-Period Dispatch of Distributed Energy Resources in Unbalanced Distribution Feeders. IEEE Trans. Power Syst 2020, 35, 2683–2692.

[28] Nazir N; Almassalkhi M Receding-Horizon Optimization of Unbalanced Distribution Systems with Time-Scale Separation for Discrete and Continuous Control Devices. In Proceedings of the 2018 Power Systems Computation Conference (PSCC), Dublin, Ireland, 11–15 June 2018; pp. 1–7.

[29] Nazir N; Almassalkhi M Stochastic multi-period optimal dispatch of energy storage in unbalanced distribution feeders. In Proceedings of the 2020 Power Systems Computation Conference (PSCC), Porto, Portugal, 29 June–3 July 2020; pp. 1–7.

[30] New York ISO. Real-Time Market LBMP - Zonal. Available online: http://mis.nyiso.com/public/P-24Alist.htm (accessed on 24 October 2020).

[31] Neukomm M; Nubbe V; Fares R Grid-interactive Efficient Buildings Technical Report Series: Overview of Research Challenges and Gaps; Technical Report; U.S. Department of Energy, Office of Energy Efficiency and Renewable Energy: Washington, DC, USA, 2019.

[32] Perfumo C; Kofman E; Braslavsky JH; Ward JK Load management: Model-based control of aggregate power for populations of thermostatcally controlled loads. Energy Convers. Manag 2012, 55, 36–48.

[33] Nandanoori SP; Kundu S; Vrabie D; Kalsi K; Lian J Prioritized threshold allocation for distributed frequency response. In Proceedings of the 2018 IEEE Conference on Control Technology and Applications (CCTA), Copenhagen, Denmark, 21–24 August 2018; pp. 237–244.

[34] Diao R; Lu S; Elizondo M; Mayhorn E; Zhang Y; Samaan N Electric water heater modeling and control strategies for demand response. In Proceedings of the 2012 IEEE Power and Energy Society General Meeting, San Diego, CA, USA, 22–26 July 2012; pp. 1–8.

[35] Kundu S; Hansen J; Lian J; Kalsi K Assessment of Optimal Flexibility in Ensemble of Frequency Responsive Loads. In Proceedings of the IEEE International Conference on Smart Grid Communication, Dresden, Germany, 23–27 October 2017.

[36] Duffaut Espinosa L; Khurram A.; Almassalkhi M A Virtual Battery Model for Packetized Energy Management. In Proceedings of the IEEE Conference on Decision and Control (to appear), Jeju Island, Korea, 14–18 December 2020.

[37] Zhou Z; Levin T; Conzelmann G Survey of US Ancillary Services Markets; Technical Report; Argonne National Lab.: Argonne, IL, USA, 2016.

[38] Coffrin C; Bent R; Sundar K; Ng Y; Lubin M PowerModels.jl: An Open-Source Framework for Exploring Power Flow Formulations. In Proceedings of the 2018 Power Systems Computation Conference (PSCC), Dublin, Ireland, 11–15 June 2018; pp. 1–8.

[39] Nazir N; Almassalkhi M Voltage positioning using co-optimization of controllable grid assets. arXiv 2019, arXiv:1911.00338.

[40] Kron G Tensor Analysis of Networks; J. Wiley & Sons, Incorporated: New York, NY, USA, 1939.

[41] Blumsack S; Hines P; Patel M; Barrows C; Sanchez EC Defining power network zones from measures of electrical distance. In Proceedings of the 2009 IEEE Power Energy Society General Meeting, Calgary, AB, Canada, 26–30 July 2009; pp. 1–8.

[42] Dorfler F; Bullo F Kron reduction of graphs with applications to electrical networks. IEEE Trans. Circuits Syst. I Regul. Pap 2012, 60, 150–163.

[43] Shukla SR; Paudyal S; Almassalkhi MR Efficient Distribution System Optimal Power Flow with Discrete Control of Load Tap Changers. IEEE Trans. Power Syst 2019, 34, 2970–2979.

[44] Christakou K; LeBoudec J; Paolone M; Tomozei D Efficient Computation of Sensitivity Coefficients of Node Voltages and Line Currents in Unbalanced Radial Electrical Distribution Networks. IEEE Trans. Smart Grid 2013, 4, 741–750.

[45] Amini M; Almassalkhi M Optimal Corrective Dispatch of Uncertain Virtual Energy Storage Systems. IEEE Trans. Smart Grid 2020, 11, 4155–4166.

[46] Garifi K; Baker K; Christensen D; Touri B Convex Relaxation of Grid-Connected Energy Storage System Models With Complementarity Constraints in DC OPF. IEEE Trans. Smart Grid 2020, 11, 4070–4079.

[47] UMBRELLA-F97-Project. Toolbox for Common Forecasting, Risk Assessment, and Operational Optimisation in Grid Security Cooperations of Transmission System Operators(TSOs). 2015. Available online: http://www.e-umbrella.eu (accessed on 19 November 2020).

[48] Haupt SE; Kosovic B; Jensen T; Lee J; Jimenez P; Lazo J; Cowie J; Mccandless T; Pearson J; Weiner G; The Sun4cast^®^ Solar Power Forecasting System: The Results of the Public-Private-Academic Partnership to Advance Solar Power Forecasting; No. NCAR/TN-526+STR; National Center for Atmospheric Research: Boulder, CO, USA, 2016; pp. 1–287.

[49] Perez R; Schlemmer J; Hemker K; Kivalov S; Kankiewicz A; Dise J Solar energy forecast validation for extended areas & economic impact of forecast accuracy. In Proceedings of the 2016 IEEE 43rd Photovoltaic Specialists Conference (PVSC), Portland, OR, USA, 5–10 June 2016; pp. 1119–1124.

[50] Bing J; Krishnani P; Bartholomy O; Hoff T; Perez R Solar monitoring, forecasting, and variability assessment at SMUD. In Proceedings of the World Renewable Energy Forum, Denver, CO, USA, 13–17 May 2012.

[51] Roald L; Andersson G Chance-constrained AC optimal power flow: Reformulations and efficient algorithms. IEEE Trans. Power Syst 2017, 33, 2906–2918.

[52] Stellato B Data-Driven Chance Constrained Optimization. Master’s Thesis, ETH-Zürich, Zürich, Switzerland, 2014.

[53] Gurobi Optimization, LLC. Gurobi Optimizer Reference Manual. 2020. Available online: http://www.gurobi.com (accessed on 24 October 2020).

[54] Wächter A; Biegler LT On the implementation of an interior-point filter line-search algorithm for large-scale nonlinear programming. Math. Program 2006, 106, 25–57.

[55] HSL, A. Collection of Fortran Codes for Large-Scale Scientific Computation. 2007. Available online: http://www.hsl.rl.ac.uk (accessed on 24 October 2020).

[56] Chassin DP; Schneider K; Gerkensmeyer C GridLAB-D: An open-source power systems modeling and simulation environment. In Proceedings of the Transmission and Distribution Conference and Exposition, Chicago, IL, USA, 21–24 April 2008; pp. 1–5.

[57] Lougee-Heimer R The Common Optimization INterface for Operations Research. IBM J. Res. Dev 2003, 47, 57–66.

[58] Botkin-Levy M; Engelmann A; Mühlpfordt T; Faulwasser T; Almassalkhi M Distributed Control of Charging for Electric Vehicle Fleets under Dynamic Transformer Ratings. arXiv 2020, arXiv:eess.SY/2007.10304.

[59] IEEE. Guide for Smart Grid Interoperability of Energy Technology and Information Technology Operation with the Electric Power System (EPS), End-Use Applications, and Loads. In IEEE Std 2030–2011; IEEE: Piscataway, NJ, USA, 2011; pp. 1–126.

[60] Distributed Energy Resources Task Force. Distributed Energy Resources: Connection, Modeling and Reliability Considerations; Technical Report; North American Electric Reliability Council (NERC): Atlanta, GA, USA, 2016.

[61] Schweizer Engineering Laboratories, Inc. SEL-3530–4 Real-Time Automation Controller (RTAC) Spec Sheet. Available online: https://cms-cdn.selinc.com/assets/Literature/Product%20Literature/Data%20Sheets/3530_DS_20200224.pdf?v=20200305-193540 (accessed on 24 October 2020).

[62] Applegate CJ; Chapin SJ Requirements and Capabilities Needed for Robust DERMS Control Verification; Technical Report; Lawrence Livermore National Laboratory (LLNL): Livermore, CA, USA, 2019.

[63] Arghandeh R; Bariya M; Cotter G; Deka D; Konakalla SAR; Seyedi Y; Shasavari A Synchronized Measurements and Their Applications in Distribution Systems: An Update. Available online: https://www.naspi.org/sites/default/files/reference_documents/naspi_distt_synchro_measure_apps_20200716.pdf (accessed on 24 October 2020).

[64] Lee P The Software-Defined Power Grid Is Here. 2020. Available online: https://spectrum.ieee.org/energy/the-smarter-grid/the-softwaredefined-power-grid-is-here (accessed on 24 October 2020).

[65] Mackiewicz RE Overview of IEC 61850 and benefits. In Proceedings of the 2006 IEEE Power Engineering Society General Meeting, Montreal, QC, Canada, 8–22 June 2006; pp. 1–8.

[66] Adrah CM; Bjørnstad S; Kure Ø Fusion networking technology for IEC 61850 inter substation communication. In Proceedings of the 2017 IEEE International Conference on Smart Grid and Smart Cities (ICSGSC), Singapore, 23–26 July 2017; pp. 152–156.

[67] Aftab MA; Hussain SS; Ali I; Ustun TS IEC 61850 based substation automation system: A survey. Int. J. Electr. Power Energy Syst 2020, 120, 106008.

[68] Taft J Electric Grid Resilience and Reliability for Grid Architecture; Pacific Northwest National Laboratory (PNNL): Richland, WA, USA, 2017.

[69] Brunner C IEC 61850 for power system communication. In Proceedings of the 2008 IEEE/PES Transmission and Distribution Conference and Exposition, Bogota, Columbia, 13–15 August 2008; pp. 1–6.

[70] Guay F; Cardinal J; Lemiex E; Guerette S Digital real-time simulator using IEC 61850 communication for testing devices. In Proceedings of the CIGRE Canada Conference, Montreal, QC, Canada, 24–26 September 2012.

[71] Nazir N; Almassalkhi M Grid-aware aggregation and realtime disaggregation of distributed energy resources in radial networks. arXiv 2019, arXiv:math.OC/1907.06709.

